# The experience of financial burden for people with multimorbidity: A systematic review of qualitative research

**DOI:** 10.1111/hex.13166

**Published:** 2020-12-02

**Authors:** James Larkin, Louise Foley, Susan M. Smith, Patricia Harrington, Barbara Clyne

**Affiliations:** ^1^ HRB Centre for Primary Care Royal College of Surgeons in Ireland Dublin Ireland; ^2^ School of Psychology National University of Ireland Galway Galway Ireland; ^3^ Health Information and Quality Authority Dublin Ireland

**Keywords:** chronic disease, evidence synthesis, financial burden, health care costs, multimorbidity, non‐communicable disease, qualitative research, systematic review

## Abstract

**Background:**

Multimorbidity prevalence is increasing globally. People with multimorbidity have higher health care costs, which can create a financial burden.

**Objective:**

To synthesize qualitative research exploring experience of financial burden for people with multimorbidity.

**Search strategy:**

Six databases were searched in May 2019. A grey literature search and backward and forward citation checking were also conducted.

**Inclusion criteria:**

Studies were included if they used a qualitative design, conducted primary data collection, included references to financial burden and had at least one community‐dwelling adult participant with two or more chronic conditions.

**Data extraction and synthesis:**

Screening and critical appraisal were conducted by two reviewers independently. One reviewer extracted data from the results section; this was checked by a second reviewer. GRADE‐CERQual was used to summarize the certainty of the evidence. Data were analysed using thematic synthesis.

**Main results:**

Forty‐six studies from six continents were included. Four themes were generated: the high costs people with multimorbidity experience, the coping strategies they use to manage these costs, and the negative effect of both these on their well‐being. Health insurance and government supports determine the manageability and level of costs experienced.

**Discussion:**

Financial burden has a negative effect on people with multimorbidity. Continuity of care and an awareness of the impact of financial burden of multimorbidity amongst policymakers and health care providers may partially address the issue.

**Patient or public contribution:**

Results were presented to a panel of people with multimorbidity to check whether the language and themes ‘resonated’ with their experiences.

## 1 INTRODUCTION

Chronic disease, particularly multimorbidity (presence of two or more chronic diseases in a person), is one of the biggest challenges for health care systems globally.^1^ The challenge of multimorbidity is primarily driven by the single‐disease focus of health care systems, clinical guidelines and research.^2^, ^3^, ^4^, ^5^ The estimated prevalence of multimorbidity in the general population ranges from 13% to 72% depending on setting and age group studied^6^ and has been increasing in recent decades.^7^, ^8^, ^9^

Multimorbidity leads to greater health care utilization due to the extra health care needs associated with having additional conditions, but also the issues that arise as a result of the interactions between these conditions.^10^ This is the primary cause of one of the central challenges of multimorbidity: the financial cost to health systems, society and the people who have multimorbidity. A systematic review of multimorbidity cost‐of‐illness studies concluded that multimorbidity was always associated with higher out‐of‐pocket (OOP) costs compared with ‘non‐multimorbidity’.^11^ Multimorbidity has also been found to be associated with between five and ten times higher OOP costs for medications than no chronic conditions.^12^ These high costs raise equity concerns, as multimorbidity disproportionately affects people from lower socioeconomic groups.^13^

The World Bank reports that every country in the world imposes some form of OOP payments for health care on people^14^ and these have been increasing.^15^ People across the world also incur other costs when accessing health care (eg transport costs) and may experience indirect costs such as reduced income from employment due to treatment‐related absenteeism. Multimorbidity is also a significant issue in low‐ and middle‐income countries (LMICs), albeit prevalence is not as high.^16^ A systematic review^17^ concludes that financial catastrophe due to non‐communicable diseases is evident across all continents and across all income strata. However, citizens of LMICs are more vulnerable to impoverishment due to OOP payments.^18^

Along with impoverishment, the financial burden associated with multimorbidity can have other negative effects including reduced medication adherence due to inability to purchase medication^12^ and reduced quality of life.^19^ The financial burden associated with multimorbidity may also have an effect on health care utilization and contribute to the higher levels of mortality^20^ and morbidity people with multimorbidity experience.

Many studies have examined costs associated with multimorbidity.^11^ However, these primarily examine the cost of multimorbidity to the health system and not the cost to the individual.^11^, ^21^ Given that people's experiences are considered key in evaluating the quality of health care,^22^, ^23^ by synthesizing many qualitative studies, people with multimorbidity can be given a greater voice^24^ and their experience of financial burden can be elucidated. The authors therefore aimed to synthesize qualitative research exploring experience of financial burden for people with multimorbidity.

## 2 METHOD

### 2.1 Study design

A systematic review of qualitative studies using thematic synthesis examining the experience of financial burden for people with multimorbidity was conducted, in order to provide a comprehensive picture of people's experiences to inform discourse and decision making.^25^ A protocol detailing the methods^26^ for this review was published. The review was registered with the International Prospective Register of Systematic Reviews (PROSPERO) (CRD42019135284). The completed review is reported according to the enhancing transparency in reporting the synthesis of qualitative research (ENTREQ) checklist^27^ (Appendix A).

### 2.2 Search strategy

The full search strategy is detailed in the protocol. A pre‐planned search was conducted in six databases (PubMed, EMBASE, CINAHL, PsycINFO, Applied Social Sciences Index and Abstracts, and LILACS) from inception to May 2019. A grey literature search, of websites considered relevant by the research team, was also conducted^28^ and completed on 26 November 2019. The authors contacted content experts for relevant articles. Forward citation checking was conducted using Scopus, a recommended database for forward citation checking.^29^ When the full text was not available, the corresponding author was contacted by email with one follow‐up. If no reply was received within one week of follow‐up, the study was excluded.

### 2.3 Study selection

Studies were included if they used a qualitative design, conducted primary data collection, included first‐ or second‐order references to financial burden and had at least one community‐dwelling adult (≥ 18 years) participant with two or more chronic conditions (see Table 1). Studies exploring people's experience of ‘chronic disease’, where no specific condition was the focus of the study and many of the participants had one condition only, were included. First‐ or second‐order references to financial burden were only included if it was clear that it was in relation to participants with multimorbidity. Studies that focused on people with a single specific chronic condition were excluded.

**Table 1:** Inclusion and exclusion criteria based on modified PICO^26^

PICoS	Inclusion criteria	Exclusion criteria
Population	At least one person with multimorbidity (defined as ≥2 chronic diseases) Community‐dwelling adults (≥18 years old)	Single named condition focus
Phenomenon of interest	Financial burden for people with multimorbidity	
Context	Any country Primary and secondary care	Residential health care facilities
Study type	Qualitative Original research (eg interviews or focus groups) Mixed methods	Quantitative

### 2.4 Screening

Search results were exported to EndNote X8, and duplicates were removed and then imported into Covidence. Firstly, one reviewer (JL) screened titles to remove entries clearly unrelated to the research question. Then, two reviewers (JL and LF) screened titles and abstracts independently, according to the inclusion/exclusion criteria (Table 1). Finally, full texts were reviewed independently by two reviewers (JL, LF). At both stages, disagreements were resolved through discussion. If agreement was not reached, then a third reviewer (BC or SS) decided on inclusion.

### 2.5 Data extraction

One reviewer (JL) extracted study characteristics using a pre‐specified pro forma.^28^ Extraction was cross‐checked by a second reviewer (BC). Data for the thematic synthesis were extracted from the results sections only by one reviewer (JL) and cross‐checked by a second reviewer (LF). Where studies presented views of participants other than people with multimorbidity, data were only extracted where it was clearly attributed to a participant with multimorbidity.

### 2.6 Data analysis

Data were analysed using Thomas and Harden's method of thematic synthesis,^30^ an inductive method used to draw inference based on common themes from studies with different designs and perspectives.^30^ The following three‐step process for thematic synthesis was conducted. First, one author read and re‐read the included studies while conducting line‐by‐line coding. Secondly, these codes were grouped into related areas to form descriptive themes. Thirdly, these descriptive themes were iteratively examined and compared to refine the relationship between them and to generate themes that go beyond the descriptive themes to provide new insights related to the review question (analytical themes). NVivo version 12 was used for analysis.

This three‐step process was carried out by one author (JL) and cross‐checked by a second author (BC). Direct quotations from study participants are presented in italics to distinguish them from second‐order data (author interpretations).

### 2.7 Public and Patient Involvement (PPI)

In order to increase the credibility of the findings, the results were presented to a PPI panel with experience of living with multimorbidity. While acknowledging that they are often occurring in another context, this process offered a modified form of ‘respondent validation’^31^ in allowing the PPI contributors to check whether the language and themes ‘resonated’ with their experiences.

### 2.8 Appraisal of studies

The critical appraisal skills programme (CASP) qualitative checklist^32^ was used to assess the quality of included studies. Studies were independently evaluated by two reviewers (JL and BC). Differences were resolved through discussion. Studies were not excluded or weighted based on quality appraisal.

### 2.9 Certainty of the evidence—CERQual assessment

The GRADE‐CERQual approach^33^ was used to summarize the authors’ confidence in the reviews findings. This assessment was conducted by one reviewer (JL) and double‐checked by a second reviewer (BC). Details of this process are in the protocol.^26^

### 2.10 Reflexivity

Details of the researchers personal worldviews and experiences are in the protocol.^26^ The researchers reflected on their personal worldviews and experiences throughout the research process.

### 2.11 Protocol deviations

Non‐English studies were excluded. The CERQual assessment and data extraction were cross‐checked by a second reviewer instead of being conducted in duplicate. A third reviewer did not oversee the analysis or the process of critical appraisal.

## 3 RESULTS

### 3.1 Search results

**FIGURE 1:**
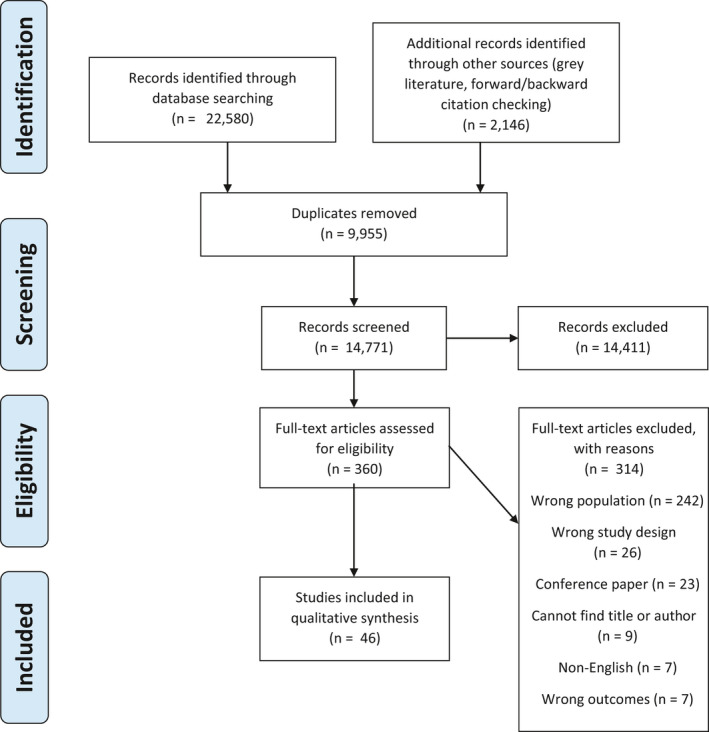
PRISMA flow chart. PRISMA, Preferred Reporting Items for Systematic Reviews and Meta‐Analyses

In total, 22,580 citations were identified from searching the databases, and 2,146 identified from grey literature searches and forward and backward citation checking of included studies. After removing 9,955 duplicates, 14,771 records were screened, of which 1,900 were excluded based on title, and 12,511 were excluded based on title and abstract. Three hundred and sixty full texts were screened and 314 were excluded (Appendix B), leaving 46 studies included for qualitative synthesis (Figure 1).

### 3.2 Characteristics of included studies

Forty‐six studies were included, two of which used the same data set.^34^, ^35^ Seven of these studies^35^, ^36^, ^37^, ^38^, ^39^, ^40^, ^41^ aimed to research financial burden or a related concept. The remaining 39 studies^34^, ^42^, ^43^, ^44^, ^45^, ^46^, ^47^, ^48^, ^49^, ^50^, ^51^, ^52^, ^53^, ^54^, ^55^, ^56^, ^57^, ^58^, ^59^, ^60^, ^61^, ^62^, ^63^, ^64^, ^65^, ^66^, ^67^, ^68^, ^69^, ^70^, ^71^, ^72^, ^73^, ^74^, ^75^, ^76^, ^77^, ^78^, ^79^ researched several issues including self‐management,^42^, ^44^, ^45^, ^46^, ^48^, ^55^, ^57^, ^60^ adherence^43^, ^52^, ^56^, ^62^, ^64^ and living with multimorbidity.^63^, ^66^, ^67^, ^70^, ^75^ There were 4,364 unique participants in total. Two studies,^73^, ^74^ used online questionnaires and included 2,689 participants, while the remaining 44 studies included 1,675 participants. From the forty‐one studies^36^, ^37^, ^38^, ^39^, ^40^, ^41^, ^42^, ^43^, ^44^, ^45^, ^46^, ^47^, ^49^, ^50^, ^51^, ^52^, ^53^, ^54^, ^55^, ^56^, ^57^, ^58^, ^59^, ^60^, ^61^, ^62^, ^63^, ^64^, ^65^, ^66^, ^67^, ^68^, ^70^, ^71^, ^72^, ^73^, ^74^, ^75^, ^76^, ^77^, ^78^, ^79^ reporting participants’ gender, 1,386 (63.3%) were male, 799 (36.5%) were female, one (0.0004%) was a transgender female and two (0.001%) were described as ‘other’. The mean age for participants was 53.6 years (from 28 studies^36^, ^37^, ^52^, ^53^, ^56^, ^59^, ^60^, ^61^, ^63^, ^65^, ^66^, ^67^, ^68^, ^71^, ^74^, ^75^, ^76^, ^77^, ^78^). The age range of participants was 20‐90 (from 15 studies^36^, ^39^, ^42^, ^44^, ^45^, ^47^, ^48^, ^50^, ^51^, ^53^, ^58^, ^61^, ^68^, ^71^, ^72^). The mean number of conditions for participants was four (from 20 studies^39^, ^41^, ^54^, ^59^, ^61^, ^62^, ^63^, ^65^, ^66^, ^71^, ^76^, ^78^, ^79^). The number of participants with multimorbidity was 2,631 (from 38 studies^34^, ^36^, ^38^, ^39^, ^40^, ^52^, ^53^, ^54^, ^55^, ^57^, ^58^, ^59^, ^60^, ^61^, ^62^, ^63^, ^65^, ^66^, ^67^, ^68^, ^70^, ^71^, ^73^, ^74^, ^75^, ^76^, ^77^, ^78^, ^79^). As outlined in the methods, some studies contained participants with a single chronic condition, but data on experience of financial burden were only extracted for participants with two or more chronic conditions.

The 46 studies were conducted in 14 different countries across six continents. Twenty‐six studies were conducted in North America,^36^, ^37^, ^53^, ^54^, ^55^, ^56^, ^62^, ^65^, ^67^, ^68^, ^69^, ^71^, ^72^, ^75^, ^76^, ^77^, ^78^, ^79^ one in South America,^41^ four in Africa,^42^, ^59^, ^60^, ^63^ four in Asia,^43^, ^57^, ^61^, ^64^ three in Europe,^48^, ^66^, ^74^ seven in Oceania^34^, ^35^, ^38^, ^39^, ^52^, ^58^, ^70^ and one study was conducted in multiple continents.^73^ Twenty‐five of the 46 included studies did not state a specific methodological approach,^35^, ^37^, ^38^, ^39^, ^54^, ^55^, ^56^, ^57^, ^58^, ^61^, ^63^, ^72^, ^73^, ^74^, ^76^, ^78^, ^79^ six used a form of phenomenology,^41^, ^59^, ^60^, ^62^, ^65^, ^70^ three used grounded theory,^36^, ^40^, ^64^ three used interpretive approaches,^34^, ^67^, ^75^ three used ethnography,^45^, ^66^, ^68^ one used narrative inquiry,^52^ one used narrative case study^53^ and one used a descriptive approach.^77^ Three studies used mixed methods,^42^, ^69^, ^71^ all of which included primary qualitative data collection. Of the 46 included studies, one was published between 1990 and 1999,^72^ seven were published between 2000 and 2009^38^, ^40^, ^44^, ^46^, ^50^, ^68^, ^75^ and 38 were published between 2010 and 2019.^34^, ^35^, ^36^, ^37^, ^47^, ^48^, ^49^, ^51^, ^52^, ^53^, ^54^, ^55^, ^56^, ^57^, ^58^, ^59^, ^60^, ^61^, ^62^, ^63^, ^64^, ^65^, ^66^, ^67^, ^69^, ^70^, ^71^, ^73^, ^74^, ^76^, ^77^, ^78^, ^79^

Twenty‐seven studies used interviews as their method of data collection,^34^, ^35^, ^36^, ^37^, ^38^, ^39^, ^40^, ^46^, ^48^, ^49^, ^50^, ^51^, ^57^, ^59^, ^60^, ^61^, ^63^, ^64^, ^65^, ^67^, ^68^, ^71^, ^72^, ^75^, ^79^ seven used focus groups,^44^, ^47^, ^58^, ^62^, ^76^, ^77^, ^78^ nine used a mix of methods,^42^, ^52^, ^53^, ^54^, ^55^, ^56^, ^66^, ^69^, ^70^ two used online questionnaires with free text sections^73^, ^74^ and one conducted ‘conversations’ with participants.^41^

Twelve studies recruited participants from primary care,^38^, ^69^, ^70^, ^71^ ten from the general population,^34^, ^35^, ^36^, ^79^ six from outpatient departments,^34^, ^51^, ^53^, ^59^, ^62^, ^66^ four from secondary/tertiary care,^52^, ^57^, ^58^, ^64^ four from other studies,^37^, ^39^, ^77^, ^78^ three from the community,^47^, ^50^, ^67^ three from ‘clinical’ settings^43^, ^44^, ^60^ and four from other settings^41^, ^42^, ^54^, ^75^ such as a chronic illness support group.^41^ The data extraction table is in Appendix E.

### 3.3 Quality appraisal

**FIGURE 2:**
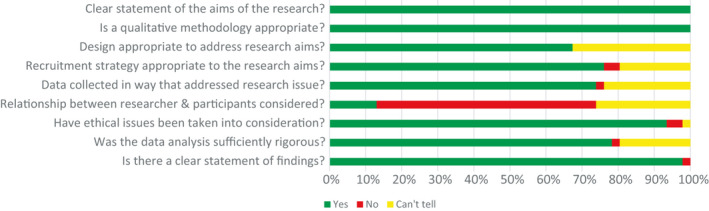
Methodological quality assessment of included studies using the Critical Appraisal Skills Programme tool

All included studies had a clear statement of the aim (see Figure 2 and Appendix C). While a qualitative methodology was appropriate in all included studies, it was unclear whether the qualitative design used was appropriate to address the research aims in 15 of the 46 studies.^38^, ^40^, ^42^, ^43^, ^48^, ^50^, ^55^, ^57^, ^63^, ^64^, ^69^, ^71^, ^73^, ^76^, ^79^ This was primarily because the researcher had not provided a justification for the design.

In 28 of the 46 included studies, there was no consideration given to the relationship between researcher and participants and the potential bias that may arise in data collection, analysis and interpretation.^34^, ^35^, ^47^, ^48^, ^49^, ^50^, ^51^, ^52^, ^55^, ^56^, ^57^, ^61^, ^64^, ^66^, ^69^, ^70^, ^71^, ^76^, ^77^, ^78^, ^79^ For 12 studies, it was unclear from the description whether there was sufficient depth of consideration given.^42^, ^68^, ^73^, ^74^

### 3.4 Certainty of the Evidence

Using the GRADE‐CERQual,^33^ certainty of the evidence for individual findings ranged from low to high (Appendix D). There was consistency in most findings across countries and settings. Confidence in review findings were downgraded primarily due to methodological limitations and relevance. The main methodological limitation was in relation to inadequate exploration of reflexivity. The main issue with relevance was the high proportion of studies from the United States.

### 3.5 Thematic synthesis

#### 3.5.1 Overview of results

Four descriptive themes related to the experience of financial burden were generated: (a) high costs, (b) access and negotiating health insurance and government supports, (c) coping strategies to manage costs and (d) reduced well‐being. Table 2 reports participant quotations that are representative of these themes and their subthemes.

**Table 2:** Representative quotations for each theme/subtheme

Theme	Representative quotation
High costs	
Direct costs	‘The financial outlay to manage these conditions appeared to be more than simply the sum of expenditures for each condition’.^68^ ‘The incidental costs of seeking healthcare, such as transportation and parking were a barrier that hindered some participants’ access to medical care. Transportation related issues included: not being able to drive independently, having to travel prohibitively long distances to healthcare facilities, costs of gasoline, and the cost of parking at hospitals and doctors’ offices’.^36^ ‘*The medications are just astronomical when it comes to money, to paying for them. I pay over, I think, $300 a month just on medications*’^51^ *‘To go to the doctor is expensive. For example, going to pick up medication at the insurance company including a round trip by taxi, and buying the medication cost a lot of money […] Everything is expensive’* ^41^
Indirect costs	‘*No, I’m not working. And, like I say, when I was working at [name of employer], I stopped taking that medicine for a whole year, trying to keep the job ‘cause I was having lotta side effects from the medicines and I couldn't do both*’.^78^ ‘The long‐term nature of this burden was aggravated by having to cease employment prematurely due to ill health. Apart from the impact that might be anticipated to result from employment loss on other domains of life, it clearly increased the financial strain associated with the management of chronic conditions’.^35^
Health insurance and government supports	
Insufficient coverage and insufficient care	*‘I rarely came to the hospital because we don't have health insurance cover for going to see doctors in the outpatient clinic here’*.^57^ ‘*Oh my God, it's awful. When I’m charged through [Medicaid managed care plan], they charge one dollar for each prescription, and I take like 20‐25 medications…plus the Lantis (insulin) and Humalog (insulin) and the syringes, the needles, the sticks, that's an extra $5 on top of the $25 I already pay…that's $30 per month, and I cannot afford that, and because of that, I’m having to pick and choose which medication to take and which medication to leave because I can't afford to buy them, and it's causing a lot of health problems*’.^40^
Safety net	‘*I'm surviving financially because of the welfare system*’^38^ ‘*[without private health insurance], you'd be out in the middle of the dead less sea*’.^34^
Complexity	‘*The fee…they said it could be like a sliding scale. But I assumed that it couldn't slide that far down for me*’.^36^ *‘For our health insurance company we are high maintenance people. So they restraint* [SIC] *us from receiving quality services such as referrals to medical specialists, treatments, procedures and diagnostic tests on time. For example, six months ago we went to the doctor for a follow up visit where we were expecting to find out how my health was but the doctor limited the visit to refilling my prescription of Morphine and nothing else’* ^41^
Coping strategies to manage costs	
Accessing informal supports	‘*There was a time that a medication was prescribed for me and that costs GH¢150.00. It was my sister's child who gave me money to purchase that medicine*’.^63^ ‘*One son kind of manages the money and lends me money when I need it and then I pay him back when I get my government [pension]*’.^67^
Making sacrifices	‘*I take my medicine only when I feel my sugar is high. Those drugs are not free you know. I pay $30 for one drug that I take for a month and I take about 9 drugs. So I don't fill them every month*’^65^ ‘*All my money goes on my health aside from basic bills. I do not buy treats, clothes, haircuts, toiletries, things for the house […] Have to spend a lot of time and energy on budgeting and I delay treatment sometimes as I have to save up*’^73^ ‘*it costs us $330 a month just for our health insurance, now that on a pension is a very very big constraint and that is why we sold our house*’.^39^
Reduced well‐being	‘*The costs are many. The drugs are expensive and sometimes I cry when I hear of the cost of the drugs*’.^59^ ‘*When you work you're whole, but when you get to the point of having to depend on other people for your income… It's like you don't become a whole person anymore. You become pieces. And if you don't have that piece to help you through that life you can't be whole. It's like you're lost*’.^36^ ‘Sheila advocated for herself but became frustrated when the physician offered her an additional prescription. The offer demonstrated to Sheila the physician's lack of care and inability to grasp her real‐life constraints: a difficult journey to the clinic and a limited income and how she could ill support a double medication fee’^75^

High costs were central to participants’ descriptions of their experiences of financial burden. Whether the costs experienced by a participant were manageable and how high these costs were was determined by the participant's level of health insurance and government supports. When health care costs were unaffordable, participants had to access informal supports or make sacrifices. Being unable to afford health care and the associated sacrifices led to a negative impact on well‐being for some participants.

### 3.6 High costs

This theme related to the scale and detail of costs associated with multimorbidity and was discussed in 36 studies. The costs were reported either in terms of direct costs (costs directly related to care such as medicines, transport and health care appointments, discussed in 32 studies) or indirect costs (costs indirectly related to care such as loss of income, discussed in 14 studies).

#### 3.6.1 Direct costs

The cost of medicines was mentioned in 23 studies^35^, ^36^, ^51^, ^56^, ^58^, ^59^, ^62^, ^63^, ^64^, ^65^, ^67^, ^68^, ^69^, ^70^, ^75^, ^76^ and appeared to take up the biggest proportion of people's resources: ‘*You kinda scrimp and save and pull all your resources together and then the cost of your medication just about gobbles that up*’.^36^ People with polypharmacy were found to have higher costs.^36^, ^39^, ^40^, ^68^, ^69^, ^75^

Cost of transportation to and from health care appointments^36^, ^58^, ^60^, ^63^, ^67^, ^69^, ^70^, ^73^, ^75^and cost of parking at health care facilities^36^, ^54^, ^58^, ^67^, ^73^ were major costs identified across studies. Participants highlighted that these issues were exacerbated when they lived long distances from health care facilities,^36^, ^58^, ^73^ when they had to attend several different clinicians^60^, ^67^, ^68^, ^73^ or when they had to return to the same health care facility several times due to lack of coordination of their care.^68^, ^73^ Two studies^54^, ^63^ highlighted that people with physical disabilities were more likely to have high transport costs as public transport was difficult to access for this group.

Health care appointments were described as a high cost repeatedly across included studies.^39^, ^41^, ^47^, ^58^, ^61^, ^65^, ^68^, ^70^, ^75^ This applied both to the cost per individual appointment and the cost associated with multiple visits. The reason for the multiple visits was sometimes polypharmacy as people needed greater monitoring and dose adjustments.^68^ Also, participants in two studies described having to pay extra due to the length of an individual consultation.^47^, ^70^

Other health care costs contributing to financial burden included purchase of aids and equipment,^37^, ^68^ dietary costs,^40^, ^58^, ^60^, ^65^, ^68^, ^73^ and costs of recommended self‐management activities^36^, ^58^, ^68^ such as gym membership.

#### 3.6.2 Indirect costs

Loss of income was the main indirect cost related to multimorbidity. Participants described how multimorbidity can lead to reduced working hours,^37^, ^39^, ^63^, ^66^, ^70^, ^72^, ^78^ early retirement,^37^, ^49^, ^70^ quitting,^35^, ^37^, ^49^, ^70^, ^72^, ^75^, ^78^ reduced promotion opportunities and earning potential,^37^ or being fired from a job.^37^, ^53^, ^62^, ^72^ These negative outcomes were attributed to three main factors. Firstly, the symptoms of the conditions such as pain, reduced energy or nausea were reported to interfere with people's ability to work.^35^, ^36^, ^37^, ^51^, ^63^, ^66^, ^78^ Secondly, some felt they were being harassed at work because of their ill health.^53^ Thirdly, the many health care appointments associated with multimorbidity,^36^, ^37^, ^51^, ^66^ which were often disjointed, necessitated time off work. Three studies highlighted the additional financial burden for participants, who have lost their jobs and no longer had access to employment‐related health insurance.^39^, ^53^, ^78^

### 3.7 Health insurance and government supports

Health insurance (private and public) and government supports often determined whether the costs participants experienced were manageable and how high these costs were. Three subthemes were generated in which health insurance and government supports affected financial burden: insufficient coverage, safety net and complexity.

#### 3.7.1 Insufficient coverage and insufficient care

Some participants could not afford care because government supports^35^, ^37^, ^39^, ^54^, ^55^, ^68^, ^73^ or health insurance ^34^, ^36^, ^37^, ^38^, ^57^, ^59^, ^63^, ^65^, ^68^, ^69^, ^70^, ^74^, ^75^, ^77^, ^78^ only partially covered health care costs, or not at all. This was particularly pronounced for medications; co‐payments for medications were reported as a financial burden, even when people had health insurance^36^, ^38^, ^40^, ^47^, ^51^, ^59^, ^63^, ^68^, ^75^ or government support.^35^, ^38^, ^39^, ^55^, ^68^

‘Even though most respondents had supplemental health insurance, often sponsored by the government or employers, only a small number of participants reported being adequately insured. Many of those who had insurance reported substantial out‐of‐pocket costs in the form of copayments’.^36^

Government supports also included social welfare payments. For some participants, social welfare payments could not cover their health care costs along with their other basic needs.^40^, ^48^, ^53^

Several participants talked about not being able to afford health insurance,^39^, ^53^ while others discussed making sacrifices (described in more detail below), including selling their home, because they felt health insurance was vital.^39^ In some cases, participants felt that their lack of health insurance and/or government supports meant they had to wait for care^41^ or could not access the best available care.^47^, ^63^

#### 3.7.2 Safety net

Some participants discussed how they could afford care because of the financial safety net that government support^36^, ^38^, ^39^, ^43^, ^52^, ^59^, ^76^ and/or health insurance^34^, ^39^, ^56^, ^68^ provided. This was described in ten studies.

The range of government supports available was wide, covering areas such as travel,^39^, ^52^ medicines,^36^, ^39^, ^43^, ^76^ consultations,^39^ emergency care^39^ and general support through pensions^38^, ^39^, ^60^ or disability payments.^60^

Health insurance was described in one study as a safety net that offered security and peace of mind.^39^ One participant described health insurance as saving them money.^39^ Another study discussed the value for money that came with having multimorbidity and purchasing health insurance:

‘several participants discuss how MM [multimorbidity] may actually facilitate CRC [colorectal cancer] screenings. Explanations were provided for this relationship—screening procedures were integrated into disease management procedures, other conditions prompted the individual to purchase insurance which then made prevention activities more economically feasible’.^45^

#### 3.7.3 Complexity

Seventeen studies outlined the financial complexities and barriers participants faced when accessing health care or reimbursement, and this applied to both government support^36^, ^52^, ^53^, ^54^, ^76^ and health insurance.^34^, ^37^, ^41^, ^45^, ^47^, ^51^, ^65^, ^68^, ^72^, ^73^, ^75^, ^77^ Participants described feeling frustrated at being just outside a certain threshold (eg age or income) set by the government^36^, ^54^ or their health insurance.^42^ In one example, there were waiver programmes available that people did not know they could access.^36^ In two studies, participants described being told that a service or medicine they were using was no longer covered by their health insurance.^51^, ^65^ In one case, there was a lack of clarity on how much a procedure was going to cost until it was carried out.^34^

These issues led participants to seek out help from health care workers,^53^ challenge the system by contacting the services responsible,^51^ protest^41^ or go to court to resolve issues.^72^

### 3.8 Coping strategies to manage costs

This theme related to coping strategies people with multimorbidity developed to manage high costs.

#### 3.8.1 Accessing informal supports

Thirteen studies mentioned help from family and friends as an informal support to manage the high costs associated with multimorbidity.^37^, ^72^ Support involved help with money,^37^, ^40^, ^53^, ^59^, ^63^, ^67^ housing,^40^, ^53^ food,^40^ managing finances,^67^ work^66^ and other areas.^40^, ^65^

##### ‘It is hard but it is my body and I have to struggle. Sometimes I ask my children for money’.^59^

There were two examples of people borrowing medicines^37^, ^69^ from family members. People also went to community resources^40^, ^58^, ^63^ such as their ‘*pastor*’^63^ for help with finances. Some participants reported having no family or friends who could help them.^40^, ^63^, ^68^

#### 3.8.2 Making sacrifices

Due to high costs, people sometimes chose, or were forced, to not adhere to a number of recommended self‐management behaviours. Cost‐related non‐adherence occurred for a very wide range of health care activities, including preventive care,^45^, ^57^, ^71^ dietary advice^40^, ^60^, ^65^ and exercise guidance.^36^, ^53^, ^65^, ^68^ Cost‐related medication non‐adherence was mentioned in 14 studies.^36^, ^39^, ^40^, ^47^, ^50^, ^52^, ^53^, ^55^, ^59^, ^62^, ^63^, ^65^, ^69^, ^70^ Polypharmacy was described as a major cause of this: ‘*I said my God, what do they think I’m a bank here or what! I have a lot of pills. […] So a few of them […] I don't take them anymore. Just can't afford it*’.^50^ Cost‐related non‐adherence even occurred for prescription medicines with very small co‐payments, due to the cumulative cost for many medicines.^40^, ^55^, ^69^ One study described a person who, due to unaffordable costs, would only take medication when they were feeling unwell or to control symptoms.^65^

High costs also led to some people not attending health care appointments.^36^, ^61^, ^65^, ^70^, ^74^ A US‐based study reported that this was more likely to occur for black people.^68^ Five studies reported that this was more likely to occur for people from deprived populations.^45^, ^61^, ^62^, ^68^, ^75^ Authors also reported that black people^68^ and people from deprived populations.^45^, ^61^, ^62^, ^68^, ^75^ were more likely to engage in cost‐related non‐adherence. Potential loss of income also led participants to choose their work over health care/self‐management.^66^, ^67^, ^78^

Not accessing health care or following guidance led to cases of adverse clinical outcomes.^36^, ^40^, ^54^, ^55^, ^65^, ^69^, ^75^ This included being hospitalized^36^, ^54^ and general exacerbation of conditions.^40^, ^55^, ^65^, ^69^, ^75^ For example, one woman with diabetes, high blood pressure, heart disease and vision impairment stated that:

‘*For every visit then, I had to pay $200… $200 every month was too much for me. So I stopped going to this doctor. This is when I started developing some problems. I mean… I mean full blown diabetes*’.^65^

Thirteen studies described people who made large personal sacrifices in order to afford their treatment.^36^, ^37^, ^39^, ^40^, ^50^, ^53^, ^54^, ^60^, ^63^, ^70^, ^72^, ^73^, ^75^ This included sacrificing necessities, such as bills,^37^, ^73^ food^36^, ^40^, ^50^, ^63^ or other needs^36^, ^39^, ^50^, ^54^, ^60^, ^73^ such as clothes. One person prioritized treatment for their condition over their family's nutrition: ‘*I used to live on noodles, home brand noodles. And my son would say; “where's the food mum?,” and I’d say, “we have to live on noodles, I need my medication”’*.^70^ Participants also discussed losing their savings,^37^, ^53^ losing their home^39^ and accruing high levels of debt in order to meet the high costs associated with multimorbidity.^53^, ^72^, ^75^

### 3.9 Reduced well‐being

Fourteen studies reported that the costs associated with multimorbidity had a negative impact on well‐being.^36^, ^37^, ^39^, ^40^, ^50^, ^53^, ^54^, ^59^, ^62^, ^63^, ^72^, ^75^, ^78^, ^79^ This manifested itself in many forms such as upset,^59^, ^63^ worry,^40^, ^59^, ^72^, ^78^ frustration^50^, ^62^, ^75^ and stress.^37^, ^54^, ^63^, ^72^ These emotions were primarily caused by people's inability to afford health care^50^, ^59^, ^62^, ^63^, ^75^, ^78^ and the associated unaffordability of necessities^37^, ^40^, ^63^:

‘it was stressful where I would have liked to have had the experience while I was convalescing to be like not worried about are my lights gonna get shut off? And sometimes that happened and it was just rough’.^37^

Not being able to work and the financial burden associated with this also had a negative impact on well‐being,^36^, ^79^ particularly on people's self‐esteem.

Financial burden left some feeling socially isolated because they could not afford social activities.^36^, ^79^ People also experienced shame and stigma because of their inability to pay for health care and the poverty they were experiencing.^36^, ^39^, ^53^, ^79^

Four studies^38^, ^39^, ^43^, ^72^ discussed people's experiences of relief at being spared financial hardship due to multimorbidity.

### 3.10 Analytical themes

Analytical themes were developed based on the descriptive themes and the research questions specified in the protocol^26^:

What are the experiences of people with multimorbidity of financial burden?

How does financial burden affect interactions between people with multimorbidity and the health care system?

How does financial burden impact on treatment burden for people with multimorbidity?

#### 3.10.1 Experiences of people with multimorbidity of financial burden

Several included papers^35^, ^36^, ^47^, ^60^, ^68^, ^70^ discuss how costs increase with increasing number of diseases (3.6 High costs): ‘Hardship was exacerbated when patients had “co‐morbidities” or “multi‐morbidities” with the cost of illness management increasing as more illnesses were being managed’.^38^ Participants described having to travel to several different clinicians, return to the same health care facility several times due to lack of coordination of their care and pay extra for longer consultations due to multiple issues. All other areas of financial burden discussed above are directly related to the costs experienced by the person, and therefore, it is likely that multimorbidity exacerbates these areas of financial burden. For example, cost‐related medication non‐adherence is highlighted as a big issue for people with polypharmacy even if the cost of each individual medication is low (3.2, 3.8.2 making sacrifices).

#### 3.10.2 The effects of financial burden on interactions between people with multimorbidity and the health care system and the effects of financial burden on treatment burden for people with multimorbidity

We used Tran's^73^ taxonomy of treatment burden to analyse the effects of financial burden on treatment burden. Treatment burden is the work of being a patient and the effect of this on the person's quality of life. Financial factors are elements of two of the components of Tran's^73^ taxonomy of treatment burden: ‘consequences of health care tasks imposed on patients’ and ‘factors that exacerbate the burden of treatment’ as well as being a central component of financial burden (3.6 High costs).

Due to the complexity of accessing financial supports some people have to use different methods to navigate the health care system. For example: people seek out help from health care workers or challenge the system to access care (3.7.3 complexity). Also, it is likely that complexities will lead to greater interactions with government agencies and insurance companies to access reimbursement or to clarify what services are available. This involves learning to navigate the health care system which is a component of treatment burden.^73^

Financial burden impacts on and interacts with another component of treatment burden: ‘health care tasks imposed on patients’.^73^ The costs of health care can sometimes create a need to avoid losing income which creates a conflict between treatment and finances and can lead some participants to choose between their treatment and their work (3.2, 3.8.2 making sacrifices). This phenomenon may be more pronounced for those with a high treatment burden as work schedules sometimes conflict with treatment regimens due to the scale and disjointed nature of health care needed (3.6 High costs).

There are indirect ways that financial burden may increase interactions with the health care system and affect treatment burden. Financial burden causes cost‐related non‐adherence, which in turn can lead to adverse clinical outcomes (3.2, 3.8.2 making sacrifices). The adverse clinical outcomes can sometimes create a need for greater health care use (3.2, 3.8.2 making sacrifices), thus creating a cycle of more interactions with the health care system (treatment burden) and financial burden.

## 4 DISCUSSION

### 4.1 Main findings

This systematic review of qualitative research identified 46 studies (45 datasets) from six continents. Financial burden was found to comprise the high direct and indirect costs associated with having multimorbidity, the coping strategies people have to use to manage these costs, and the effect of these costs and associated strategies on people's well being; government supports and health insurance often determined the manageability and levels of costs experienced. These phenomena were found to exist across settings, including settings with universal health coverage (UHC). Using the GRADE‐CERQual^33^ approach, the certainty of the evidence ranged from low to high, but was moderate for most findings.

Few studies included in the review aimed to research financial burden specifically, and amongst those that did, their characterization and definition of financial burden varied. Based on the results above, we adapted Eton's^51^ definition of treatment burden to define financial burden of multimorbidity as the monetary costs associated with having multimorbidity, the extra workload this creates and the impact these factors have on a person's functioning and well‐being.

### 4.2 In the context of other research

Many people with a chronic disease experience financial burden. However, the results of this review highlight that multimorbidity increases costs and in turn exacerbates other areas of financial burden. The manner in which multimorbidity increases costs has been found across other studies: multimorbidity is associated with greater health care utilization,^80^ higher rates of polypharmacy,^81^ fragmented care^5^, ^82^ and an increased likelihood of leaving paid employment.^83^ The results presented here provide insights into how these increased costs are experienced by people with multimorbidity.

Studies suggest that socio‐economically deprived groups with multimorbidity are more vulnerable to financial burden.^84^, ^85^ This is exemplified in the findings which showed that deprived populations were more vulnerable to cost‐related non‐adherence. Given that socio‐economically deprived groups are often found to be more likely to have multimorbidity,^13^, ^86^ interventions and resources may benefit socio‐economically deprived groups most.

The findings reinforce that financial burden compromises the health of people with multimorbidity through non‐adherence to medication and self‐management practices and non‐attendance at health care appointments, which could create a negative cycle. The components of this negative cycle have been documented in other research.^87^, ^88^, ^89^, ^90^, ^91^ The results also show that financial burden compromises the health of people with multimorbidity through its impact on well‐being, causing people to feel stressed, upset, worried and frustrated.

Despite the inclusion of countries with vastly different levels of health care access and government supports, most themes existed across countries. For example, insufficient coverage and insufficient care were evident across six continents and in countries such as Kenya^59^ and England.^48^ What may differ greatly between countries is the proportion of the population and sub‐groups within it who are vulnerable to financial burden and the degree of impact financial burden may have on these populations. Levels of poverty are greater in LMICs,^92^ and people in LMICs are more vulnerable to impoverishment due to OOP payments.^18^

There were a disproportionate number of studies included from the United States which played a role in reducing the certainty of the evidence. One theme that was primarily evident in North America was complexities of health coverage. This is consistent with evidence that the United States has a complex health financing system,^93^ though it may also be because there is a disproportionate amount of multimorbidity research from the United States.^2^

### 4.3 Implications

Despite the financial burden faced by people with multimorbidity, Patel and colleagues^94^ suggest that clinicians are unlikely to raise the issue of financial burden with patients. This may be due to the wide range of issues faced by people with multimorbidity along with short consultation times.^95^ Health care workers are often unaware of the issues people face with the treatments they are prescribed^96^, ^97^, ^98^ and have been found to focus more on patients’ biomedical information than their psychosocial information.^99^ However, there is evidence that some clinicians, such as GPs,^94^, ^100^ are aware of the financial burden associated with multimorbidity. When prescribing/recommending treatments or self‐management practices, health care workers should consider the affordability for the patient, as costs can have unintended consequences including non‐adherence, reduced well‐being and nutritional deficits. This consideration may involve referral to a social worker or welfare rights advisor^101^, ^102^ and/or a discussion with the patient about cost implications.

Given the impact of health care utilization and uncoordinated care on financial burden, interventions should be aimed at addressing these areas. Wallace and colleagues^95^ suggest that improving continuity of care by being assigned a named doctor in primary care can reduce complication rates and use of secondary care for people with multimorbidity. Also, case managers may provide an alternative or an adjunct to improve care coordination for people with multimorbidity.^103^, ^104^

It is clear from the results that people with multimorbidity experience financial burden in a range of contexts including in countries where forms of UHC exist.^48^ The ‘safety net’ discussed by participants does, however, highlight the potential of health coverage to prevent or alleviate the impacts of financial burden. The WHO define UHC as ‘ensuring that all people have access to needed health services of sufficient quality to be effective while also ensuring that the use of these services does not expose the user to financial hardship’.^105^ The WHO point out that all countries can make strides towards UHC,^106^ including coverage for sick leave and travel to/from health care appointments which were identified in this analysis as drivers of financial burden for people with multimorbidity. UHC also has the potential to remove some of the complexities associated with health coverage highlighted by this review, such as eligibility thresholds.

With regard to the implications of this study for research, the results show that financial burden has a large impact on treatment burden. The expanded Multimorbidity Treatment Burden Questionnaire^107^ includes a question on costs, but this only relates to medication and equipment, excluding other areas such as appointments and travel, which we found to be important for people with multimorbidity. Also, the questionnaire^107^ does not cover the complexities of health coverage that people with multimorbidity face.

### 4.4 Strengths and limitations

The review protocol was published^26^ and registered on PROSPERO. The review was reported according to ENTREQ guidelines.^27^ The study comprised a broad disciplinary team, which many multimorbidity studies are lacking.^21^ The search strategy was broad. Thematic synthesis was an appropriate method of data analysis as it is used for studies with ‘thin’ data and analysis.^30^ The use of PPI was novel and added to the credibility of the findings. Two reviewers for screening reduced bias. This review offers a counterbalance to the disproportionate amount of literature focussing on the cost of multimorbidity to the health system.^21^ The studies identified were relatively recent, with 38 of the 46 included studies published between 2010 and 2019; therefore, the findings are likely applicable to current care.

The exclusion of non‐English studies represents a bias. However, given that meaning may be lost in translation^108^ and that there was breadth of countries and contexts covered by included studies, it was not a significant bias. The primary search was conducted over one year ago. However, given the extent of the search strategy and the large number of studies included from a variety of contexts, the authors concluded that the addition of new studies was unlikely to substantially change the findings. Despite the broad search strategy, some studies may not have been retrieved due to difficulties accessing qualitative literature.^109^

## 5 CONCLUSION

The direct and indirect costs associated with multimorbidity are the fundamental components of financial burden for people with multimorbidity. However, this review highlights that financial burden is not simply the costs associated with multimorbidity, but also the coping strategies people use to manage costs and the negative effect both of these have on well‐being; government supports and health insurance often determined the manageability and level of costs experienced. Considering participants’ many references to insufficient health coverage, UHC has the potential to reduce financial burden for people with multimorbidity. Greater consideration amongst policymakers and health care workers of all costs associated with accessing treatments can also mitigate financial burden. Finally, greater continuity of care can increase care coordination and reduce health care utilization, thus reducing financial burden for people with multimorbidity.

## CONFLICT OF INTEREST

The authors declare no conflicts of interest.

## AUTHOR CONTRIBUTIONS

James Larkin contributed to conceptualization, methodology, critical appraisal, data analysis, screening and writing of the original draft. Louise Foley contributed to review and editing, screening and data extraction. Susan M. Smith contributed to conceptualization, funding acquisition, methodology, project administration, supervision, and review and editing. Patricia Harrington contributed to conceptualization, methodology, supervision, and review and editing. Barbara Clyne contributed to conceptualization, methodology, critical appraisal, data analysis, supervision, and review and editing.

## Supporting information

Appendix A

Appendix B

Appendix C

Appendix D

Appendix E

## Data Availability

The data that support the findings of this study are available in the supplementary material of this article.
