# Supplementary material for: The experience of financial burden for people with multimorbidity: A systematic review of qualitative research
Source: Health Expect. 2020 Dec 2;24(2):282–95. doi: 10.1111/hex.13166 (PMC8077119; doi:10.1111/hex.13166)
Supplement: Supplementary file 2 — Appendix B [file HEX-24-282-s002.docx]

| **Reason for exclusion** |  |
| --- | --- |
| Wrong population (n=242) | ^1-242^ |
| Wrong study design (n=26) | ^243-268^ |
| Conference paper (n=23) | ^269-291^ |
| Cannot find title or author (n=9) | ^292-300^ |
| Non-english (n=7) | ^301-307^ |
| Wrong outcomes (n=7) | ^308-314^ |

1. Abbott S, Hobby L. Poverty and health: Primary care patients living at the interface. *Health Education Journal.* 2005;64(4):363-371.

2. Abel S, Whitehead LC, Coppell KJ. Making dietary changes following a diagnosis of prediabetes: a qualitative exploration of barriers and facilitators. *Diabetic Medicine.* 2018;35(12):1693-1699.

3. Aebischer Perone S, Nikolic R, Lazic R, et al. Addressing the needs of terminally-ill patients in Bosnia-Herzegovina: Patients' perceptions and expectations. *BMC Palliative Care.* 2018;17(1).

4. Ajayi IO, Jegede AS, Falade CO, Sommerfeld J. Assessing resources for implementing a community directed intervention (CDI) strategy in delivering multiple health interventions in urban poor communities in Southwestern Nigeria: A qualitative study. *Infectious Diseases of Poverty.* 2013;2(1):1-18.

5. Aji B, Yamamoto SS, Sauerborn R. The economic impact of the insured patients with severe chronic and acute illnesses: a qualitative approach. *Glob Health Action.* 2014;7:22526.

6. Allen S, Carlson G. Psychosocial themes in durable employment transitions. *Work.* 2003;20(3):185-197.

7. Al-Rousan T, Schwabkey Z, Jirmanus L, Nelson BD. Health needs and priorities of Syrian refugees in camps and urban settings in Jordan: perspectives of refugees and health care providers. *Eastern Mediterranean Health Journal.* 2018;24(3):243-253.

8. Amurwon J, Hajdu F, Yiga DB, Seeley J. "Helping my neighbour is like giving a loan…" -the role of social relations in chronic illness in rural Uganda. *BMC Health Services Research.* 2017;17:1-12.

9. Anderson JM, Blue C, Holbrook A, Ng M. On chronic illness: immigrant women in Canada's work force--a feminist perspective. *Canadian Journal of Nursing Research.* 1993;25(2):7-22.

10. Angel Hor‐Yan L, Kuang Z, Carrie Ho‐Kwan Y, Ayub S, Eng‐Kiong Y. Vouchers for primary healthcare services in an ageing world? The perspectives of elderly voucher recipients in Hong Kong. *Health & Social Care in the Community.* 2018;26(3):374-382.

11. Ansah JP, Islam AM, Koh V, et al. Systems modelling as an approach for understanding and building consensus on non-communicable diseases (NCD) management in Cambodia. *BMC Health Services Research.* 2019;19(1):N.PAG-N.PAG.

12. Armstrong N, Herbert G, Brewster L. Contextual barriers to implementation in primary care: an ethnographic study of a programme to improve chronic kidney disease care. *Family Practice.* 2016;33(4):426-431.

13. Atinga RA, Yarney L, Gavu NM. Factors influencing long-term medication non-adherence among diabetes and hypertensive patients in Ghana: A qualitative investigation. *PLoS ONE.* 2018;13(3):e0193995.

14. Audulv A, Asplund K, Norbergh K-G. The Integration of Chronic Illness Self-Management. *Qualitative Health Research.* 2012;22(3):332-345.

15. Azami‑Aghdash S, Ghojazadeh M, Hossein Aghaei M, Naghavi‑Behzad M, Asgarlo Z. Perspective of Patients, Patients' Families, and Healthcare Providers Towards Designing and Delivering Hospice Care Services in a Middle Income Country. *Indian Journal of Palliative Care.* 2015;21(3):341-348.

16. Badger TA, Gagan MJ, McNiece C. Community analysis for health planning with vulnerable populations. *Clinical Nurse Specialist: The Journal for Advanced Nursing Practice.* 2001;15(3):95-102.

17. Bates MJ, Chitani A, Dreyer G. Palliative care needs of patients living with end-stage kidney disease not treated with renal replacement therapy: An exploratory qualitative study from Blantyre, Malawi. *Afr.* 2017;9(1):e1-e6.

18. Baxter K. Changing choices: disabled and chronically ill people's experiences of reconsidering choices. *Chronic Illness.* 2013;9(2):116-132.

19. Becetti K, Robbins L, Mancuso C, Gordon JK, Spiera RF. Perceived barriers to mental health care among patients with systemic sclerosis: A qualitative study. *Arthritis and Rheumatology.* 2017;69.

20. Becker M, Jaspers B, King C, Radbruch L, Voltz R, Nauck F. Did you seek assistance for writing your advance directive? A qualitative study. *Wiener Klinische Wochenschrift.* 2010;122(21-22):620-625.

21. Beckerman NL. Living With Lupus: A Qualitative Report. *Social Work in Health Care.* 2011;50(4):330.

22. Bedell A, Taft T, Craven MR, Guadagnoli L, Hirano I, Gonsalves N. Impact on Health-Related Quality of Life in Adults with Eosinophilic Gastritis and Gastroenteritis: A Qualitative Assessment. *Digestive Diseases & Sciences.* 2018;63(5):1148-1157.

23. Bergeron CD, Friedman DB, Sisson DC, et al. Awareness, Perceptions, and Communication Needs About the Affordable Care Act Across the Life Span. *American Journal of Health Education.* 2016;47(2):108-116.

24. Bezon J. *An ethnographic approach to health needs assessment of rural elderly*, UNIVERSITY OF SOUTH FLORIDA; 1992.

25. Binswanger IA, Nowels C, Corsi KF, et al. Return to drug use and overdose after release from prison: a qualitative study of risk and protective factors. *NIDA Addiction Science & Clinical Practice.* 2012;7:1-9.

26. Birungi J, Luzze C, Etukoit BM, Mwesigwa R. Given little attention, the views of HIV positive patients on fixed drug combinations - Taso Uganda experience. *American Journal of Hypertension.* 2009;22:5.

27. Bouchelle Z, Rawlins Y, Hill C, Bennet J, Perez LX, Oriol N. Preventative health, diversity, and inclusion: a qualitative study of client experience aboard a mobile health clinic in Boston, Massachusetts. *International Journal for Equity in Health.* 2017;16:1-10.

28. Brandt R, Polinsky C, Katz J, et al. Factors influencing risk reduction surgery among BRCA mutation carriers: A qualitative analysis. *Journal of Clinical Oncology.* 2012;30(27).

29. Brataas HV, Evensen AE. Life stories of people on sick leave from work because of mild mental illness, pain and fatigue. *Work.* 2015;53(2):285-291.

30. Brekke E, Lien L, Davidson L, Biong S. First-person experiences of recovery in co-occurring mental health and substance use conditions. *Advances in Dual Diagnosis.* 2017;10(1):13-24.

31. Brod M. ADHD burden of illness in older adults: A life course perspective in the US. *International Psychogeriatrics.* 2013;25:S49-S50.

32. Broerse JEW, Bunders JFG, Caron-Flinterman JF, Teerling J. Patients' priorities concerning health research: the case of asthma and COPD research in the Netherlands. *Health Expectations.* 2005;8(3):253-263.

33. Bryant L, Martini N, Chan J, et al. Could the polypill improve adherence? The patient perspective. *Journal of Primary Health Care.* 2013;5(1):28-35.

34. Burnett K, Glaspole I, Holland AE. Understanding the patient's experience of care in idiopathic pulmonary fibrosis. *Respirology.* 2017;22:167.

35. Camargo Plazas Mdel P, Cameron BL. Using Freire's Participatory Educational Method to Understand the Experience of Living With Chronic Illness in the Current Age of Globalization. *J Nurs Res.* 2015;23(2):83-93.

36. Cameron KL. *Older homeless women with depression*, University of Arizona; 2010.

37. Canaway R, Manderson L, Oldenburg B. Perceptions of benefit of complementary therapy use among people with diabetes and cardiovascular disease. *Forsch Komplementarmed.* 2014;21(1):25-33.

38. Capoccia V, Croze C, Cohen M, O'Brien JP. Sustaining enrollment in health insurance for vulnerable populations: lessons from massachusetts. *Psychiatric Services.* 2013;64(4):360-365.

39. Capp R, Kelley L, Ellis P, et al. Reasons for Frequent Emergency Department Use by Medicaid Enrollees: A Qualitative Study. *Academic Emergency Medicine.* 2016;23(4):476-481.

40. Carney MT, Finkelstein R, Block D, et al. An opportunity to innovate: The aging of eastern queens and nassau county. *Journal of the American Geriatrics Society.* 2015;63:S68.

41. Chandler JT. *Care-Seeking Experiences of Undocumented, Mexican Immigrant Women with Chronic Illness: A Phenomenological Study*, University of California, San Francisco; 2011.

42. Chatterjee A, Yu EJ, Tishberg L. Exploring opioid use disorder, its impact, and treatment among individuals experiencing homelessness as part of a family. *Drug and Alcohol Dependence.* 2018;188:161.

43. Chen J, Jing X, Liu X, et al. Assessment of factors affecting diabetes management in the City Changing Diabetes (CCD) study in Tianjin. *PLoS ONE.* 2019;14(2).

44. Chileshe M, Bond VA. Barriers and outcomes: TB patients co-infected with HIV accessing antiretroviral therapy in rural Zambia. *AIDS Care.* 2010;22:51-59.

45. Choi J, Kushner KE, Mill J, Lai DWL. Health Experiences of Korean Immigrant Women in Retirement. *Health Care for Women International.* 2014;35(11/12):1317-1338.

46. Chubon SJ, Schulz RM, Lingle EW, Jr., Coster-Schulz MA. Too many medications, too little money: how do patients cope? *Public Health Nursing.* 1994;11(6):412-415.

47. Chuma J, Gilson L, Molyneux C. Treatment-seeking behaviour, cost burdens and coping strategies among rural and urban households in Coastal Kenya: an equity analysis. *Tropical Medicine & International Health.* 2007;12(5):673-686.

48. Coelho SP, Costa T, Barbosa MM, Capelas ML, De Mello RA, Sa L. Palliative home care, for a holistic approach to the patient and family. *Palliative Medicine.* 2016;30(6):NP294.

49. Cummingham PJ, Miller C, Cassil A. Living on the edge: health care expenses strain family budgets. *Res Briefs.* 2008(10):1-14.

50. Dalvi V, Nandakumar M. Patient non-adherence: an interpretative phenomenological analysis. *International Journal of Health Care Quality Assurance.* 2017;30(3):274-284.

51. Dassieu L, Kaboré JL, Choinière M, Arruda N, Roy É. Chronic pain management among people who use drugs: A health policy challenge in the context of the opioid crisis. *International Journal of Drug Policy.* 2019.

52. Davenport A, Stark S, Quian A, Sheyn D, Mangel J. 15: Identifying barriers to third-line therapy: a patient-centered approach to refractory overactive bladder. *American Journal of Obstetrics and Gynecology.* 2019;220(3):S716.

53. Daveson BA, Harding R, Shipman C, et al. The real-world problem of care coordination: A longitudinal qualitative study with patients living with advanced progressive illness and their unpaid caregivers. *PLoS ONE.* 2014;9(5).

54. de Carvalho Conceição AIC, Marinho CLA, Costa JR, Silva RSd, Lira GG. PERCEPTIONS OF CHRONIC KIDNEY PATIENTS IN THE REFUSAL OF THE KIDNEY TRANSPLANTATION. *Journal of Nursing UFPE / Revista de Enfermagem UFPE.* 2019;13(3):664-673.

55. DeGrezia MG. *Exploration of coping strategies in older, community dwelling, HIV positive individuals in baltimore*, ProQuest Information & Learning; 2013.

56. DeGrezia MGPRNA, Scrandis DPCP-BC. Successful Coping in Urban, Community-Dwelling Older Adults With HIV. *The Journal of the Association of Nurses in AIDS Care.* 2015;26(2):151.

57. Delea S, Buckley C, Hanrahan A, McGreal G, Desmond D, McHugh S. Management of diabetic foot disease and amputation in the Irish health system: a qualitative study of patients' attitudes and experiences with health services. *BMC Health Services Research.* 2015;15(1):251-251.

58. DiGiacomo M, Green A, Rodrigues E, Mulligan K, Davidson PM. Developing a gender-based approach to chronic conditions and women's health: A qualitative investigation of communitydwelling women and service provider perspectives. *BMC Women's Health.* 2015;15(1).

59. Drastrup AM, Mogensen KH, Almarsdottir AB, Arevalo LC, Kamper AL. Medication adherence to antihypertensives and lipid-lowering drugs in relation to beliefs about medicine among chronic kidney disease patients: A mixed methods study. *Nephrology Dialysis Transplantation.* 2018;33:i157.

60. Drumm RD, McBride DC, Metsch L, Page JB, Dickerson K, Jones B. "The rock always comes first": drug users' accounts about using formal health care. *Journal of Psychoactive Drugs.* 2003;35(4):461-469.

61. Enriquez M, Moormeier J, Lafferty W. The management of chronic diseases in rural Missouri practices. *Mo Med.* 2012;109(3):210-215.

62. Fletcher CN, Winter M, Shih A-T. Tracking the transition from welfare to work. *Journal of Sociology and Social Welfare.* 2008;35(3):115-132.

63. Foo ASC, Lee TW, Soh CR. Discrepancies in end-of-life decisions between elderly patients and their named surrogates. *Annals of the Academy of Medicine Singapore.* 2012;41(4):141-153.

64. Fossey E, Harvey C, Mokhtari M, Meadows G. Self-Rated Assessment of Needs for Mental Health Care: A Qualitative Analysis. *Community Mental Health Journal.* 2012;48(4):407-419.

65. Foster JM, McDonald VM, Guo M, Reddel H. Adherence barriers, facilitators and resilience in patients with severe asthma: A qualitative study. *American Journal of Respiratory and Critical Care Medicine.* 2017;195.

66. Foster MM, Mitchell GK. 'The onus is on me': primary care patient views of Medicare-funded team care in chronic disease management in Australia. *Health Expectations.* 2015;18(5):879-891.

67. Fraeyman J, Symons L, De Loof H, et al. Medicine price awareness in chronic patients in Belgium. *Health Policy.* 2015;119(2):217-223.

68. Freij M, Weiss L, Gass J, et al. 'Just Like I'm Saving Money in the Bank': Client Perspectives on Care Coordination Services. *Journal of Gerontological Social Work.* 2011;54(7):731-748.

69. Ganguli I, Thompson RW, Ferris TG. What can five high cost patients teach us about healthcare spending? *Healthcare.* 2017;5(4):204-213.

70. Garthwaite K. Becoming incapacitated? Long-term sickness benefit recipients and the construction of stigma and identity narratives. *Sociology of Health & Illness.* 2015;37(1):1-13.

71. Gebremariam MK, Bjune GA, Frich JC. Barriers and facilitators of adherence to TB treatment in patients on concomitant TB and HIV treatment: a qualitative study. *BMC Public Health.* 2010;10(1):651-651.

72. Glasser I, Wang F, Reardon J, et al. Improving COPD Care in a Medically Underserved Primary Care Clinic: A Qualitative Study of Patient Perspectives. *Copd.* 2016;13(5):616-621.

73. Goldberg E, Beitz JM. The lived experience of diverse elders with chronic wounds. *Ostomy Wound Management.* 2010;56(11):36-46.

74. Goldsmith LJ, Kolhatkar A, Popowich D, Holbrook AM, Morgan SG, Law MR. Understanding the patient experience of cost-related non-adherence to prescription medications through typology development and application. *Social Science and Medicine.* 2017;194:51-59.

75. Gott M, Allen R, Moeke-Maxwell T, Gardiner C, Robinson J. 'No matter what the cost': A qualitative study of the financial costs faced by family and whnau caregivers within a palliative care context. *Palliative Medicine.* 2015;29(6):518-528.

76. Goudge J, Gilson L, Russell S, et al. Affordability, availability and acceptability barriers to health care for the chronically ill: longitudinal case studies from South Africa. *BMC Health Services Research.* 2009;9:75-75.

77. Govender V, Fried J, Birch S, Chimbindi N, Cleary S. Disability Grant: a precarious lifeline for HIV/AIDS patients in South Africa. *BMC Health Services Research.* 2015;15(1):227-227.

78. Gowani A, Ahmed HI, Khalid W, et al. Facilitators and barriers to NCD prevention in Pakistanis-invincibility or inevitability: a qualitative research study. *BMC Res Notes.* 2016;9:282.

79. Grabois E, Young ME. Managed care experiences of persons with disabilities. *Journal of Rehabilitation.* 2001;67(3):13-19.

80. Grant SJ, Smith CA, Hunter J, et al. Supportive care and integrative oncology: Barriers and unmet needs. *Asia-Pacific Journal of Clinical Oncology.* 2017;13:214.

81. Graves BA, Hamner K, Nikles S, Wells H. Use of Community-Based Participatory Research toward Eliminating Rural Health Disparities. *Online Journal of Rural Nursing & Health Care.* 2015;15(2):63-87.

82. Grudzen C, Stone S, Mohanty S, et al. “I want to be taking my own last breath”: Patients' reflections on illness when presenting to an emergency department at the end of life (723). *Journal of Pain and Symptom Management.* 2011;41(1):285-286.

83. Guilcher SJT, Hamilton-Wright S, Skinner W, et al. "Talk with me": perspectives on services for men with problem gambling and housing instability. *BMC Health Services Research.* 2016;16:1-13.

84. Haddy CA, Ward HM, Angley MT, McKinnon RA. Consumers' views of pharmacogenetics--A qualitative study. *Res Social Adm Pharm.* 2010;6(3):221-231.

85. Haghgoo HA, Haji Ahmad T. Structural factors involve in the process of family adaptation to disorders in activities of daily living in stroke survivors. *Cerebrovascular Diseases.* 2016;41:12.

86. Hall BJ, Garabiles MR, Latkin CA. Work life, relationship, and policy determinants of health and well-being among Filipino domestic Workers in China: a qualitative study. *BMC Public Health.* 2019;19(1):N.PAG-N.PAG.

87. Hall JP, LaPierre TA, Kurth NK. Oral Health Needs and Experiences of Medicaid Enrollees With Serious Mental Illness. *American Journal of Preventive Medicine.* 2018;55(4):470-479.

88. Hamed R, Tariah HA, Hawamdeh ZM. Personal factors affecting the daily functioning and well-being of patients with multiple sclerosis using the international classification of functioning model: A qualitative study. *International Journal of Mental Health.* 2012;41(4):47-61.

89. Haque MA, Budi A, Azam Malik A, Suzanne Yamamoto S, Louis VR, Sauerborn R. Health coping strategies of the people vulnerable to climate change in a resource-poor rural setting in Bangladesh. *BMC public health.* 2013;13:565.

90. Hauff AJ. *Medical respite care for Fargo-Moorhead homeless population: A needs assessment*, North Dakota State University; 2013.

91. Hedman A, Lindqvist E, Nygård L. How older adults with mild cognitive impairment relate to technology as part of present and future everyday life: a qualitative study. *BMC Geriatrics.* 2016;16:1-12.

92. Hewitt-Taylor J, Bond C, Hean S, Barker S. The experiences of older people who live with a long-term condition. *Nursing Older People.* 2013;25(6):21-25.

93. Higashi RT, Craddock Lee SJ, Leonard T, Cuate EL, Cole J, Pruitt SL. Multiple Comorbidities and Interest in Research Participation Among Clients of a Nonprofit Food Distribution Site. *Clin Transl Sci.* 2015;8(5):584-590.

94. Hills H. Changing pathways to care and issues of comorbidity in persons with Opioid Dependence in Florida. *Heroin Addiction and Related Clinical Problems.* 2016;18(3):43.

95. Hinder S, Greenhalgh T. "This does my head in". Ethnographic study of self-management by people with diabetes. *BMC Health Services Research.* 2012;12(1):83-83.

96. Hovick SR, Johnson-Turbes CA, Freimuth VS, et al. Assessing perceptions of and responses to multiple health risks among the southern poor. *Preventing Chronic Disease.* 2011;8(1):A11-A11.

97. Hung SL, Fu SN, Lau PS, Wong SY. A qualitative study on why did the poorly-educated Chinese elderly fail to attend nurse-led case manager clinic and how to facilitate their attendance. *International Journal for Equity in Health.* 2015;14:10.

98. Hunter J, Ussher J, Parton C, et al. Australian integrative oncology services: a mixed-method study exploring the views of cancer survivors. *BMC Complementary & Alternative Medicine.* 2018;18(1):1-14.

99. Hunter WG, Hesson A, Davis JK, et al. Patient-physician discussions about costs: definitions and impact on cost conversation incidence estimates. *BMC health services research.* 2016;16:108.

100. Jeal N, Salisbury C, Turner K. The multiplicity and interdependency of factors influencing the health of street-based sex workers: A qualitative study. *Sexually Transmitted Infections.* 2008;84(5):381-385.

101. Jennens HR, Ramasamy R, Tenni B. Reasons for default from treatment of chronic illnesses in a primary healthcare program in rural Tamil Nadu. *Indian J Public Health.* 2013;57(3):173-176.

102. Jerant AF, von Friederichs-Fitzwater MM, Moore M. Patients' perceived barriers to active self-management of chronic conditions. *Patient Education & Counseling.* 2005;57(3):300-307.

103. Jerome D, Dehail P, Daviet JC, et al. Stroke in under-75-year-olds: expectations, concerns and needs. *Ann Phys Rehabil Med.* 2009;52(7-8):525-537.

104. Jirojwong S, Manderson L. Physical health and preventive health behaviors among Thai women in Brisbane, Australia. *Health Care for Women International.* 2002;23(2):197-206.

105. Johansson KA, Miljeteig I, Kigwangalla H, Norheim OF. HIV priorities and health distributions in a rural region in Tanzania: a qualitative study. *Journal of Medical Ethics.* 2011;37(4):221-226.

106. Johnson LP, Asigbee FM, Crowell R, Negrini A. Pre-surgical, surgical and post-surgical experiences of weight loss surgery patients: a closer look at social determinants of health. *Clin.* 2018;8(4):265-274.

107. Joseph-Kent KE. *Autism spectrum disorders and the healthcare experiences of aging adults*, ProQuest Information & Learning; 2018.

108. Kabir A, Maitrot MRL. Exploring the effects of health shocks on anti-poverty interventions: Experience of poor beneficiary households in Bangladesh. *Cogent Medicine.* 2018;5(1):1-14.

109. Karampli E, Triga E, Kyriopoulos J, Athanasakis K, Tsiantou V. Views of physicians and patients with chronic conditions on generic medicines in Greece after the introduction of measures to promote their consumption: Findings from a qualitative study. *GaBI Journal.* 2016;5(1):9-20.

110. Kaur H, Kaur H, Venkateashan M. Perceived Family Support and Quality of Life of Elderly Population. *International Journal of Nursing Education.* 2015;7(4):91-97.

111. Keating A, Lee AL, Holland AE. Lack of perceived benefit and inadequate transport influence uptake and completion of pulmonary rehabilitation in people with chronic obstructive pulmonary disease: a qualitative study. *J Physiother.* 2011;57(3):183-190.

112. Kennaugh R, Byles J, Tavener M. Beyond widowhood: Do prior discovered themes that describe the experiences of older Australian widowed women persist over time? *Women & Health.* 2016;56(7):827-842.

113. Khoza SR, Kortenbout W. An investigation of compliance in type II diabetic patients attending clinic at Church of Scotland Hospital. *Curationis.* 1995;18(4):10-14.

114. Komaric N, Bedford S, van Driel ML. Two sides of the coin: patient and provider perceptions of health care delivery to patients from culturally and linguistically diverse backgrounds. *BMC Health Services Research.* 2012;12:322.

115. Koolwal P, Madrigal L, Rothstein M, Barnoya J. Identifying perceptions of chronic kidney disease in a hemodialysis population in Guatemala. *Annals of Global Health.* 2016;82(3):577.

116. Kristiansen M, Kessing LL, Norredam M, Krasnik A. Migrants' perceptions of aging in Denmark and attitudes towards remigration: findings from a qualitative study. *BMC Health Services Research.* 2015;15(1):225-225.

117. Kuluski K, Bensimon CM, Alvaro C, Lyons RF, Schaink AK, Tobias R. LIFE INTERRUPTED: THE IMPACT OF COMPLEX CHRONIC DISEASE FROM THE PERSPECTIVE OF HOSPITALIZED PATIENTS. *Illness, Crisis & Loss.* 2014;22(2):127-144.

118. Laba T-L, Lehnbom E, Brien J-A, Jan S. Understanding if, how and why non-adherent decisions are made in an Australian community sample: a key to sustaining medication adherence in chronic disease? *Res Social Adm Pharm.* 2015;11(2):154-162.

119. Lansbury G. Chronic pain management: a qualitative study of elderly people's preferred coping strategies and barriers to management. *Disability & Rehabilitation.* 2000;22(1-2):2-14.

120. LaPierre TA, Zimmerman MK, Hall JP. “Paying the price to get there”: Motherhood and the dynamics of pregnancy deliberations among women with disabilities. *Disability and Health Journal.* 2017;10(3):419-425.

121. Lattof SR. Health insurance and care-seeking behaviours of female migrants in Accra, Ghana. *Health policy and planning.* 2018;33(4):505-515.

122. Latypov A, Otiashvili D, Zule W. Drug scene, drug use and drug-related health consequences and responses in Kulob and Khorog, Tajikistan. *International Journal of Drug Policy.* 2014;25(6):1204-1214.

123. Lauvergeon S, Burnand B, Peytremann-Bridevaux I. Chronic disease management: a qualitative study investigating the barriers, facilitators and incentives perceived by Swiss healthcare stakeholders. *BMC Health Services Research.* 2012;12(1):176-176.

124. Li L, Sloan DH, Mehta AK, Willis G, Weaver MS, Berger AC. Life perceptions of patients receiving palliative care and experiencing psycho-social-spiritual healing. *Ann.* 2017;6(3):211-219.

125. Lineback CM, Mervak CM, Revels SL, Kemp MT, Reddy RM. Barriers to Accessing Optimal Esophageal Cancer Care for Socioeconomically Disadvantaged Patients. *Annals of Thoracic Surgery.* 2017;103(2):416-421.

126. Liu X, Chen J, Volkmann AM, et al. Assessment of diabetes vulnerability in the City Changing Diabetes (CCD) study in Tianjin. *Diabetes/Metabolism Research and Reviews.* 2016;32:76-77.

127. Lohman MC, Whiteman KL, Yeomans FE, Cherico SA, Christ WR. Qualitative Analysis of Resources and Barriers Related to Treatment of Borderline Personality Disorder in the United States. *Psychiatric Services.* 2017;68(2):167-172.

128. Longman JM, Rix E, Johnston JJ, Passey ME. Ambulatory care sensitive chronic conditions: what can we learn from patients about the role of primary health care in preventing admissions? *Australian Journal of Primary Health.* 2018;24(4):304-310.

129. Lu H, Wang W, Xu L, et al. Healthcare seeking behaviour among Chinese elderly. *International Journal of Health Care Quality Assurance.* 2017;30(3):248-259.

130. Lutz BJ, Hall AG, Vanhille SB, et al. A Framework Illustrating Care-Seeking Among Older Adults in a Hospital Emergency Department. *Gerontologist.* 2018;58(5):942-952.

131. Ma S, Shi J, Li L. Dilemmas in caring for older adults in Zhejiang Province, China: a qualitative study. *BMC Public Health.* 2019;19(1):N.PAG-N.PAG.

132. Macha J, Kuwawenaruwa A, Makawia S, Mtei G, Borghi J. Determinants of community health fund membership in Tanzania: a mixed methods analysis. *BMC Health Services Research.* 2014;14(1):538-538.

133. Maertens JA. *Barriers to nutrition management among people living with HIV on antiretroviral therapy*, ProQuest Information & Learning; 2012.

134. Mahomed R, Patterson E, St John W. Factors Influencing Possible Participation in Chronic Disease Self-management Courses. *Australian Journal of Primary Health - Interchange.* 2008;14(3):19-26.

135. Mahon G, O'Brien B, O'Conor L. The experience of chronic illness among a group of Irish patients: A qualitative study. *Journal of Research in Nursing.* 2014;19(4):330-342.

136. Malone RE. *The almshouse revisited: Heavy users of emergency services*, ProQuest Information & Learning; 1996.

137. Maneze D, Dennis S, Chen H-Y, et al. Multidisciplinary care: experience of patients with complex needs. *Australian Journal of Primary Health - Interchange.* 2014;20(1):20-26.

138. Manski-Nankervis J-A, Furler J, Audehm R, Blackberry I, Young D. Potentially preventable hospitalisations: are they a useful marker of access to and experience of care in general practice among people with type 2 diabetes? *Australian Journal of Primary Health.* 2015;21(2):214-220.

139. Maree JE, Jansen Van Rensburg JJM, Van Rensburg JJMJ. Suitability of quality-of-life outcome measures in palliative care in the South African setting. *Palliative & Supportive Care.* 2016;14(2):118-128.

140. Marlow E, White MC, Chesla CA. Barriers and facilitators: parolees' perceptions of community health care. *Journal of Correctional Health Care.* 2010;16(1):17-26.

141. Martinez IL, Carter-Pokras O. Assessing Health Concerns and Barriers in a Heterogeneous Latino Community. *Journal of Health Care for the Poor and Underserved.* 2006;17(4):899-909.

142. Mataure P, Thupayagale-Tshweneagae GB. EXPECTATIONS OF CLIENTS ENROLLED IN A COMMUNITY AND HOME-BASED CARE PROGRAMME: INSIGHTS FROM ZIMBABWE. *Africa Journal of Nursing & Midwifery.* 2013;15(1):149-162.

143. Matheson L, Nayoan J, Rivas C, et al. Conceptualising dimensions of adjustment and self-management to advanced prostate cancer-A qualitative study. *Psycho-Oncology.* 2019;28:3-4.

144. Mavhu W, Dauya E, Bandason T, et al. Chronic cough and its association with TB-HIV co-infection: factors affecting help-seeking behaviour in Harare, Zimbabwe. *Tropical Medicine & International Health.* 2010;15(5):574-579.

145. Mayston R, Lloyd-Sherlock P, Gallardo S, et al. A journey without maps — Understanding the costs of caring for dependent older people in Nigeria, China, Mexico and Peru. *PLoS ONE.* 2017;12(8).

146. McElfish PA, Balli ML, Hudson JS, et al. Identifying and Understanding Barriers and Facilitators to Medication Adherence Among Marshallese Adults in Arkansas. *Journal of Pharmacy Technology.* 2018;34(5):204-215.

147. McIlfatrick S. Assessing palliative care needs: Views of patients, informal carers and healthcare professionals. *Journal of Advanced Nursing.* 2007;57(1):77-86.

148. Mead H, Andres E, Katch H, Siegel B, Regenstein M. Gender differences in psychosocial issues affecting low-income, underserved patients' ability to manage cardiovascular disease. *Womens Health Issues.* 2010;20(5):308-315.

149. Mead H, Andres E, Regenstein M. Underserved Patients' Perspectives on Patient-Centered Primary Care: Does the Patient-Centered Medical Home Model Meet Their Needs? *Medical Care Research and Review.* 2014;71(1):61-84.

150. Mei H, Turale S. Coping experience of health concerns and physical disability for older Chinese people: A qualitative, descriptive study. *Nursing & Health Sciences.* 2017;19(4):444-451.

151. Mendenhall E, Omondi GB, Bosire E, et al. Stress, diabetes, and infection: Syndemic suffering at an urban Kenyan hospital. *Social Science & Medicine.* 2015;146:11-20.

152. Mercado-Martinez FJ, Levin-Echeverri R. [Care for chronic kidney disease in Uruguay: the perspective of kidney transplant patients]. *Cadernos de Saude Publica.* 2017;33(10):e00160416.

153. Metta E, Bailey A, Kessy F, Geubbels E, Haisma H. Illness experiences of diabetes in the context of malaria in settings experiencing double burden of disease in southeastern Tanzania. *PLoS ONE.* 2017;12(5):e0178394.

154. Mikhail BI, Ragheb MS. Health-related concerns and experiences of employed perimenopausal women in Alexandria, Egypt. *Health Care for Women International.* 1996;17(2):173-186.

155. Miller B, Holland A, Tierney A, Norris N, King S, Shalit N. Influences on dietary intake in adults with chronic obstructive pulmonary disease: A qualitative study. *Respirology.* 2015;20:130.

156. Mirza M, Luna R, Mathews B, Hasnain R, et al. Barriers to Healthcare Access Among Refugees with Disabilities and Chronic Health Conditions Resettled in the US Midwest. *Journal of Immigrant and Minority Health.* 2014;16(4):733-742.

157. Motta-Ochoa R, Bertrand K, Flores-Aranda J, et al. A qualitative study of addiction help-seeking in people with different co-occurring mental disorders and substance use problems. *International Journal of Mental Health and Addiction.* 2017;15(4):883-899.

158. Munce SE, Webster F, Fehlings MG, et al. Perceived facilitators and barriers to self-management in individuals with traumatic spinal cord injury: a qualitative descriptive study. *BMC Neurology.* 2014;14(1):48-48.

159. Nabhani JA, Vargas G, Kwan L, et al. The experience of low-income men with prostate cancer transitioning from disease-specific coverage to comprehensive insurance under the affordable care act. *Journal of Cancer Policy.* 2018;18:33-39.

160. Nelms L, Johnson V, Teshuva K, Foreman P, Stanley J. Social and Health Factors Affecting Community Service Use by Vulnerable Older People. *Australian Social Work.* 2009;62(4):507-524.

161. Nickasch B, Marnocha SK. Healthcare experiences of the homeless. *Journal of the American Academy of Nurse Practitioners.* 2009;21(1):39-46.

162. Nogueira TE, Dias DR, Rios LF, Silva ALM, Jordão LMR, Leles CR. Perceptions and experiences of patients following treatment with single-implant mandibular overdentures: A qualitative study. *Clinical oral implants research.* 2019;30(1):79-89.

163. O'Keeffe J, Long SK, Liu K, Kerr M. How do they manage? Disabled elderly persons in the community who are not receiving Medicaid long-term care services. *Home Health Care Services Quarterly.* 2001;20(4):73-90.

164. Oliveira Chagas V, Carvalho Vila VdS, Pandolfo Provin M, Goreti Amaral R, Severino Pereira M. ACCESS TO MEDICATION: THE PERSPECTIVES OF PEOPLE WHO RESORT TO JUDICIAL PROCEEDINGS. *Ciencia, Cuidado e Saude.* 2016;15(3):489-497.

165. Onyango MA, Vian T, Hirsch I, et al. Perceptions of Kenyan adults on access to medicines for non-communicable diseases: A qualitative study. *PLoS ONE.* 2018;13(8):e0201917.

166. Orzech KM, Vivian J, Huebner Torres C, Armin J, Shaw SJ. Diet and Exercise Adherence and Practices Among Medically Underserved Patients With Chronic Disease: Variation Across Four Ethnic Groups. *Health Education & Behavior.* 2013;40(1):56-66.

167. Oshi DC, Oshi SN, Alobu IN, Ukwaja KN. GENDER-RELATED FACTORS INFLUENCING WOMEN'S HEALTH SEEKING FOR TUBERCULOSIS CARE IN EBONYI STATE, NIGERIA. *Journal of Biosocial Science.* 2016;48(1):37-50.

168. Overshon S. *Assessing patient perceptions of self-care: Examining chronic disease in an underserved rural area*, ProQuest Information & Learning; 2018.

169. Padgett DK, Henwood B, Abrams C, Drake RE. Social relationships among persons who have experienced serious mental illness, substance abuse, and homelessness: Implications for recovery. *Am J Orthopsychiatry.* 2008;78(3):333-339.

170. Pattenden JF, Roberts H, Lewin RJP. Living with heart failure; patient and carer perspectives. *European Journal of Cardiovascular Nursing.* 2007;6(4):273-279.

171. Perez-Leon S, Pesantes MA, Aya Pastrana N, Raman S, Miranda J, Suggs LS. Food Perceptions and Dietary Changes for Chronic Condition Management in Rural Peru: Insights for Health Promotion. *Nutrients.* 2018;10(11):1563.

172. Persoon S, Buffart LM, Chinapaw MJM, et al. Return to work experiences of patients treated with stem cell transplantation for a hematologic malignancy. *Supportive Care in Cancer.* 2018.

173. Peter Van E. Urban elderly with chronic illness: local understandings and emerging discrepancies in North Sulawesi, Indonesia. *Anthropology and Medicine.* 2003;10(3):325-341.

174. Petroka K, Campbell-Bussiere R, Dychtwald DK, Milliron BJ. Barriers and facilitators to healthy eating and disease self-management among older adults residing in subsidized housing. *Nutr Health.* 2017;23(3):167-175.

175. Portacolone E, Covinsky K, Rubinstein R, Ortez-Alfaro J, Halpern J, Johnson J. PRIORITIES AND CONCERNS OF OLDER AFRICAN AMERICANS LIVING ALONE WITH COGNITIVE IMPAIRMENT: AN IN-DEPTH PERSPECTIVE. *Alzheimer's and Dementia.* 2018;14(7):P1423.

176. Posselt M, McDonald K, Procter N, de Crespigny C, Galletly C. Improving the provision of services to young people from refugee backgrounds with comorbid mental health and substance use problems: addressing the barriers. *BMC Public Health.* 2017;17:1-17.

177. Potter CM, Kelly L, Hunter C, Fitzpatrick R, Peters M. The context of coping: a qualitative exploration of underlying inequalities that influence health services support for people living with long‐term conditions. *Sociology of Health & Illness.* 2018;40(1):130-145.

178. Price JA, Soares AI, Asante AD, Martins JS, Williams K, Wiseman VL. "I go I die, I stay I die, better to stay and die in my house": understanding the barriers to accessing health care in Timor-Leste. *BMC health services research.* 2016;16(1):535.

179. Rammant E, Fonteyne V, Decaestecker K, et al. Understanding physical activity behavior in patients with bladder cancer before and after radical cystectomy: a qualitative interview study. *Clinical Rehabilitation.* 2019;33(4):750-761.

180. Rat AC, Stewart A, Rogers P, Mosher DP, Lacaille D. Challenges and barriers to employment that persons with osteoarthritis face at work due to their condition. *Arthritis and Rheumatology.* 2018;70:297-298.

181. Reid EA, Gudina EK, Ayers N, Tigineh W, Azmera YM. Caring for Life-Limiting Illness in Ethiopia: A Mixed-Methods Assessment of Outpatient Palliative Care Needs. *Journal of Palliative Medicine.* 2018;21(5):622-630.

182. Rice K, Webster F. Care interrupted: Poverty, in-migration, and primary care in rural resource towns. *Social Science & Medicine.* 2017;191:77-83.

183. Rifkin DE, Laws MB, Rao M, et al. Medication adherence behavior and priorities among older adults with CKD: a semistructured interview study. *American Journal of Kidney Diseases.* 2010;56(3):439-446.

184. Rogers C, Johnson J, Nueslein B, Edmunds D, Valdez RS. "I Love Fruit But I Can't Afford It": Using Participatory Action Research to Develop Community-Based Initiatives to Mitigate Challenges to Chronic Disease Management in an African American Community Living in Public Housing. *Journal of racial and ethnic health disparities.* 2018;5(6):1315-1327.

185. Röttger J, Blümel M, Fuchs S, Busse R. Assessing the responsiveness of chronic disease care—Is the World Health Organization’s concept of health system responsiveness applicable? *Social Science & Medicine.* 2014;113:87-94.

186. Royster MO, Richmond A, Eng E, Margolis L. Hey Brother, How's Your Health? A Focus Group Analysis of the Health and Health-Related Concerns of African American Men in a Southern City in the United States. *Men and Masculinities.* 2006;8(4):389-404.

187. Saine ME, Barg FK, Carbonari DM, Gross R, Re VL. Barriers and facilitators to initiating and completing direct-acting antiviral therapy for chronic hepatitis C infection. *Pharmacoepidemiology and Drug Safety.* 2018;27:480.

188. Salway S, Platt L, Harriss K, Chowbey P. Long-term health conditions and Disability Living Allowance: exploring ethnic differences and similarities in access. *Sociology of Health & Illness.* 2007;29(6):907-930.

189. Sansom D. *Vulnerable individuals with diabetes navigating the health care system: A grounded theory study*, ProQuest Information & Learning; 2014.

190. Saunders L, Newman S, Aust R, Krause JS. Qualitative study of barriers and facilitators to cigarette smoking after spinal cord injury. *Rehabilitation Psychology.* 2018;63(3):400-407.

191. Sav A, McMillan SS, Kelly F, et al. The ideal healthcare: priorities of people with chronic conditions and their carers. *BMC Health Services Research.* 2015;15:1-10.

192. Schafheutle EI, Hassell K, Noyce PR, Weiss MC. Access to medicines: cost as an influence on the views and behavior of patients. *Health & Social Care in the Community.* 2002;10(3):187-195.

193. Schafheutle EI, Schafheutle EI. Patients' views on the UK policy of prescription charges-Insights from qualitative interviews. *Res Social Adm Pharm.* 2008;4(4):343-354.

194. Schepens Niemiec SL, Carlson M, Martinez J, Guzman L, Mahajan A, Clark F. Developing Occupation-Based Preventive Programs for Late-Middle-Aged Latino Patients in Safety-Net Health Systems. *American Journal of Occupational Therapy.* 2015;69(6):1-11.

195. Schlosser AV, Joshi K, Smith S, Thornton AB, Trapl E, Bolen S. The promises and perils of a produce prescription program: A qualitative exploration. *Journal of General Internal Medicine.* 2017;32(2):S349.

196. Selten EM, Vriezekolk JE, Geenen R, et al. Reasons for Treatment Choices in Knee and Hip Osteoarthritis: A Qualitative Study. *Arthritis Care & Research.* 2016;68(9):1260-1267.

197. Seto E, Leonard KJ, Cafazzo JA, Masino C, Barnsley J, Ross HJ. Self-care and Quality of Life of Heart Failure Patients at a Multidisciplinary Heart Function Clinic. *Journal of Cardiovascular Nursing.* 2011;26(5):377-385.

198. Sevilla-Cazes J, Bowles KH, Ahmed FS, et al. A qualitative study of patient-reported challenges to heart failure home management. *Circulation: Cardiovascular Quality and Outcomes.* 2017;10.

199. Sewell K, Andreae S, Luke E, Safford MM. Perceptions of and barriers to use of generic medications in a rural African American population, Alabama, 2011. *Preventing Chronic Disease.* 2012;9:E142-E142.

200. Shanmugam IS. Problems of long term hospitalization. *International Journal of Rehabilitation Research.* 1982;5(4):549-550.

201. Singh JA. Gout and comorbidity: a nominal group study of people with gout. *Arthritis Res Ther.* 2017;19(1):204.

202. Singleton JK. Caring for themselves: facilitators and barriers to women home care workers who are chronically ill following their care plan. *Health Care for Women International.* 2002;23(6/7):692-702.

203. Skogens L, Ninive von G, Topor A. Initiating and maintaining a recovery process – experiences of persons with dual diagnosis. *Advances in Dual Diagnosis.* 2018;11(3):101-113.

204. Sloane PD, Cohen LW, Haac BE, Zimmerman S. Health care experiences of U.S. retirees living in Mexico and Panama: a qualitative study. *BMC Health Services Research.* 2013;13:411.

205. Smith SK, Johnston J, Rutherford C, Hollowell R, Tanabe P. Identifying Social-Behavioral Health Needs of Adults with Sickle Cell Disease in the Emergency Department: JEN JEN. *Journal of Emergency Nursing.* 2017;43(5):444-450.

206. Stanhope V, Henwood BF. Activating People to Address Their Health Care Needs: Learning from People with Lived Experience of Chronic Illnesses. *Community Mental Health Journal.* 2014;50(6):656-663.

207. Steckowych K, Smith M, Stevens A, Spiggle S, Li H. Building the case: Changing consumer perceptions of the value of expanded community pharmacist services. *Journal of the American Pharmacists Association.* 2018;58(3):e111-e112.

208. Suma TK, Shenoy RK, Kumaraswami V. A qualitative study of the perceptions, practices and socio-psychological suffering related to chronic brugian filariasis in Kerala, southern India. *Ann Trop Med Parasitol.* 2003;97(8):839-845.

209. Suttiratana S, Naranjo D. Transition-aged latino & black youth: Experiences with T1DM management. *Diabetes.* 2015;64:A57.

210. Suurmond J, Rosenmöller DL, el Mesbahi H, Lamkaddem M, Essink-Bot M-L. Barriers in access to home care services among ethnic minority and Dutch elderly—A qualitative study. *International Journal of Nursing Studies.* 2016;54:23-35.

211. Tan ST, Quek RYC, Haldane V, et al. The social determinants of chronic disease management: perspectives of elderly patients with hypertension from low socio-economic background in Singapore. *International Journal for Equity in Health.* 2019;18(1):N.PAG-N.PAG.

212. Tan SYMSW, Yong LMOM, Foong JYEB, Wong NHSM, Chew LLBA, Koh YLMM. Securing and Sustaining Employment: Concerns of HIV Patients in Singapore. *Social Work in Health Care.* 2013;52(10):881.

213. Thorpe O, Kumar S, Johnston K. Barriers to and enablers of physical activity in patients with COPD following a hospital admission: a qualitative study. *Int J Chron Obstruct Pulmon Dis.* 2014;9:115-128.

214. Tierney S, Deaton C, Elwers H, et al. Physical activity as heart failure therapy: Patient perspectives. *European Journal of Cardiovascular Nursing.* 2011;10:S26.

215. Timpel P, Lang C, Wens J, et al. Individualising Chronic Care Management by Analysing Patients' Needs -- A Mixed Method Approach. *International Journal of Integrated Care (IJIC).* 2017;17(5):1-12.

216. Tjia J, Givens JL, Karlawish JH, Okoli-Umeweni A, Barg FK. Beneath the surface: discovering the unvoiced concerns of older adults with Type 2 diabetes mellitus. *Health Education Research.* 2008;23(1):40-52.

217. Tlili F, Tinsa F, Skhiri A, Zaman S, Phillimore P, Ben Romdhane H. Living with diabetes and hypertension in Tunisia: popular perspectives on biomedical treatment. *International Journal of Public Health.* 2015;60:31-37.

218. Turner BJ, Craig K, Makanji VS, Flores BE, Hernandez L. Improving support and education of low‐income baby boomers diagnosed with chronic hepatitis C virus infection through universal screening. *Journal of Clinical Nursing.* 2017;26(23-24):4605-4612.

219. Vaccher S, Kannangara D, Baysari M, et al. Identifying barriers to treating gout effectively: A qualitative study of patients' and general practitioners' understanding and management of gout. *Internal Medicine Journal.* 2015;45:14.

220. van Dijk-de Vries A, Moser A, Mertens VC, van der Linden J, van der Weijden T, van Eijk JT. The ideal of biopsychosocial chronic care: how to make it real? A qualitative study among Dutch stakeholders. *BMC Family Practice.* 2012;13:14.

221. Van Durme T, Macq J, Anthierens S, et al. Stakeholders' perception on the organization of chronic care: a SWOT analysis to draft avenues for health care reforms. *BMC Health Services Research.* 2014;14:179.

222. Vassilev I, Rogers A, Sanders C, et al. Social status and living with a chronic illness: an exploration of assessment and meaning attributed to work and employment. *Chronic Illness.* 2014;10(4):273-290.

223. Veronese S, Gallo G, Valle A, et al. The palliative care needs of people severely affected by neurodegenerative disorders: A qualitative study. *Progress in Palliative Care.* 2015;23(6):331-342.

224. Walden EL. An Exploration of the Experience of Lesbians with Chronic Illness. *Journal of Homosexuality.* 2009;56(5):548.

225. Walters V, Charles N. 'I just cope from day to day': Unpredictability and anxiety in the lives of women. *Social Science & Medicine.* 1997;45(11):1729-1739.

226. Weinert C, Whitney AL, Hill W, Cudney S. Chronically ill rural women's views of health care. *Online Journal of Rural Nursing & Health Care.* 2005;5(2):18p-18p.

227. Wheeler AJ, Roennfeldt H, Slattery M, Krinks R, Stewart V. Codesigned recommendations for increasing engagement in structured physical activity for people with serious mental health problems in Australia. *Health & Social Care in the Community.* 2018;26(6):860-870.

228. Whittemore R, Dixon J. Chronic illness: the process of integration. *Journal of Clinical Nursing.* 2008;17(7B):177-187.

229. Widar M, Ahlstrom G, Ek AC. Health-related quality of life in persons with long-term pain after a stroke. *Journal of Clinical Nursing.* 2004;13(4):497-505.

230. Williams SJ, Bury MR. Impairment, disability and handicap in chronic respiratory illness. *Social Science and Medicine.* 1989;29(1989):609-616.

231. Wilson C, O'Malley AS, Bozzolo C, McCall N, Ma S. Patient Experiences with Chronic Care Management Services and Fees: a Qualitative Study. *JGIM: Journal of General Internal Medicine.* 2019;34(2):250-255.

232. Wilson KS, Silberberg MR, Brown AJ, Yaggy SD. Health needs and barriers to healthcare of women who have experienced intimate partner violence. *Journal of Women's Health (15409996).* 2007;16(10):1485-1498.

233. Winsett RP. Facilitated advance care planning in end stage renal disease positively enhanced patients' hope. *Evidence Based Nursing.* 2007;10(2):64-64.

234. Winton MB. HEALTH, HEALTH PERCEPTIONS, HEALTHCARE PRACTICES, AND INFLUENCING FACTORS AMONG KOREAN IMMIGRANTS LIVING IN RURAL TEXAS. *Health, Health Perceptions, Healthcare Practices & Influencing Factors Among Korean Immigrants Living in Rural Texas.* 2014:1-1.

235. Wong ST, Regan S. Patient perspectives on primary health care in rural communities: effects of geography on access, continuity and efficiency. *Rural & Remote Health.* 2009;9(1):1142-1142.

236. Yadav UN, Chandrasekharan V, Guddattu V, Gruiskens JRJH. Mixed method approach for determining factors associated with late presentation to HIV/AIDS care in southern India. *Journal of Postgraduate Medicine.* 2016;62(3):173-177.

237. Hincapie AL, Gupta V, Brown SA, Metzger AHJJopp. Exploring perceived barriers to medication adherence and the use of mobile technology in underserved patients with chronic conditions. 2019;32(2):147-153.

238. Yen L, Gillespie J, Rn Y-HJ, et al. Health professionals, patients and chronic illness policy: a qualitative study. *Health Expectations.* 2011;14(1):10-20.

239. Youmans SL, Schillinger D, Mamary E, Stewart A. Older African Americans' perceptions of pharmacists. *Ethnicity & Disease.* 2007;17(2):284-290.

240. Zeng M, Nicholas D, Marlinga J, et al. Barriers and facilitators to diabetes care: Qualitative study. *Canadian Family Physician.* 2017;63(2):S97.

241. Zuurmond M, Mactaggart I, Kannuri N, Murthy G, Oye JE, Polack S. Barriers and facilitators to accessing health services: A qualitative study amongst people with disabilities in Cameroon and India. *International Journal of Environmental Research and Public Health.* 2019;16(7).

242. Restorick Roberts A, Betts Adams K, Beckette Warner CJJow, aging. Effects of chronic illness on daily life and barriers to self-care for older women: A mixed-methods exploration. 2017;29(2):126-136.

243. Andersen M, Tinsley J, Milfort D, et al. HIV health care access issues for women living with HIV, mental illness, and substance abuse. *AIDS Patient Care & STDs.* 2005;19(7):449-459.

244. Brand FN, Smith RT. Medical care and compliance among the elderly after hospitalization. *The International Journal of Aging & Human Development.* 1975;5(4):331-346.

245. Chapman SL, Hall JP, Moore JM. Health care access affects attitudes about health outcomes and decisions to apply for social security disability benefits. *Journal of Disability Policy Studies.* 2013;24(2):113-121.

246. Dunn K, Torres A, Tiscani J. Functional status outcomes in a quality of life study with Latinas. *Journal of Multicultural Nursing & Health (JMCNH).* 2004;10(2):39-47.

247. Eggermont N, Van Damme W, Van Olmen J. Evaluation of a peer-education program for diabetes and hypertension in rural Cambodia. *Tropical Medicine and International Health.* 2011;16:343-344.

248. Elskamp AB, Hartholt KA, Patka P, van Beeck EF, van der Cammen TJ. Why older people refuse to participate in falls prevention trials: a qualitative study. *Exp Gerontol.* 2012;47(4):342-345.

249. Gajewski BJ, Leenerts MH, Smith CE. A systematically tested intervention for managing reactive depression. *Nursing Research.* 2003;52(6):401-409.

250. Garner SL. *A retrospective study of sleep breathing disorder therapies and treatment attitudes among adult heart failure patients*, ProQuest Information & Learning; 2013.

251. Graffam J, Shinkfield AJ. The Life Conditions of Australian Ex-Prisoners: An Analysis of Intrapersonal, Subsistence, and Support Conditions. *International Journal of Offender Therapy and Comparative Criminology.* 2012;56(6):897.

252. Hall JP, Carroll SL, Moore JM. Health Care Behaviors and Decision-Making Processes Among Enrollees in a State High-Risk Insurance Pool: Focus Group Findings. *American Journal of Health Promotion.* 2010;24(5):304-310.

253. Hossain WA, Ehtesham MW, Salzman GA, Jenson R, Calkins CF. Healthcare access and disparities in chronic medical conditions in urban populations. *South Med J.* 2013;106(4):246-254.

254. Lu JF, Chen CM, Chi MJ. Advocacy for home telehealth care among the pre-elderly with chronic disease in Taiwan. *International Journal of Nursing Practice.* 2012;18:87.

255. Manak M, Ratcliff C, Thyle A, Duomai S. Poverty Reduction in India through Palliative Care: A Pilot Project. *Indian Journal of Palliative Care.* 2017;23(1):41-45.

256. McIntyre I, Broughen C, Trepman E, Embil JM. Foot and ankle problems of Aboriginal and non-Aboriginal diabetic patients with end-stage renal disease. *Foot & Ankle International.* 2007;28(6):674-686.

257. McNamara RJ, McKeough ZJ, Mo LR, Dallimore JT, Dennis SM. Community-based exercise training for people with chronic respiratory and chronic cardiac disease: a mixed-methods evaluation. *Int J Chron Obstruct Pulmon Dis.* 2016;11:2839-2850.

258. Olmos-Ochoa TT, Niv N, Hellemann G, et al. Barriers to participation in web-based and in-person weight management interventions for serious mental illness. *Psychiatric Rehabilitation Journal.* 2019.

259. Portz JD, Bayliss EA, Bull S, et al. Using the Technology Acceptance Model to Explore User Experience, Intent to Use, and Use Behavior of a Patient Portal Among Older Adults With Multiple Chronic Conditions: Descriptive Qualitative Study. *Journal of Medical Internet Research.* 2019;21(4):N.PAG-N.PAG.

260. Radhakrishnan K, Bo X, Jacelon CS. Unsustainable HomeTelehealth: A Texas Qualitative Study. *Gerontologist.* 2016;56(5):830-840.

261. Rahimpour M, Lovell NH, Celler BG, McCormick J. Patients' perceptions of a home telecare system. *International Journal of Medical Informatics.* 2008;77(7):486-498.

262. Russell D, Oberlink MR, Shah S, Evans L, Bassuk K. Addressing the Health and Wellness Needs of Vulnerable Rockaway Residents in the Wake of Hurricane Sandy: Findings From a Health Coaching and Community Health Worker Program. *Journal of Public Health Management & Practice.* 2018;24(2):137-145.

263. Rutten-van Mölken M, Leijten F, Hoedemakers M, et al. Strengthening the evidence-base of integrated care for people with multi-morbidity in Europe using Multi-Criteria Decision Analysis (MCDA). *BMC Health Services Research.* 2018;18(1):N.PAG-N.PAG.

264. Suzuki R, Krahn G, Small E, Peterson-Besse J. Multi-level Barriers to Obtaining Mammograms for Women with Mobility Limitations: Post Workshop Evaluation. *American Journal of Health Behavior.* 2013;37(5):711-718.

265. Villarreal YR, Torres LR, Stotts A, Ren Y, Sampson M, Bordnick PS. Stress, Depression, and Drug Use Among Aging Mexican American Men Living in the Barrio. *Journal of Social Work Practice in the Addictions.* 2017;17(4):388-401.

266. Wildman JM, Moffatt S, Steer M, Laing K, Penn L, O'Brien N. Service-users' perspectives of link worker social prescribing: a qualitative follow-up study. *BMC Public Health.* 2019;19(1):N.PAG-N.PAG.

267. Yeager VA, Wharton MK, Monnette A, et al. Non-Face-to-Face Chronic Care Management: A Qualitative Study Assessing the Implementation of a New CMS Reimbursement Strategy. *Population Health Management.* 2018;21(6):454-461.

268. Young AS, Sullivan G, Bogart LM, Koegel P, Kanouse DE. Needs for services reported by adults with severe mental illness and HIV. *Psychiatric Services.* 2005;56(1):99-101.

269. Abara E, Reid C, Abara N, Lacroix M, Thomas C. Interprofessional teleurology care in rural Northern Ontario: Lessons from patients and relatives. *Canadian Urological Association Journal.* 2018;12(9):S205.

270. Apesoa-Varano EC, Barker JC, Unützer J, Aguilar-Gaxiola S, Hinton L. “Me siento inutil:” Masculinity and depression among older Mexican men. *American Journal of Geriatric Psychiatry.* 2011;19(3):S93-S94.

271. Buccoliero L, Magri M, Bellio E, Solinas E. A marketing approach to innovation in chronic care: new value for patients in Lombardian region. *International Journal of Integrated Care (IJIC).* 2017;17(3):29-30.

272. Chhabra M, Ho JJ, Chaiyachati K, et al. Transitions of care at a community hospital: A series of qualitative interviews. *Journal of General Internal Medicine.* 2013;28:S224.

273. Doan CT, Ramirez-Zohfeld V, Ferreira MR, et al. Rationale, facilitators, and barriers to colorectal cancer screening among a racial and ethnic diverse population. *Journal of General Internal Medicine.* 2015;30:S244.

274. Dong R, Leung C, Naert M, et al. Chronic disease stigma, skepticism of the health system, and socio-economic fragility: Factors impacting receptiveness to a non-communicable disease intervention in rural kenya. *Circulation.* 2018;138.

275. Gill A, Kuluski K, Peckham A, et al. Functional Limitations Experienced by Older Adults with Complex Care Needs and Its Impact on Access to Community Based Health and Social Care. *International Journal of Integrated Care (IJIC).* 2017;17:1-2.

276. Ginting TT. Contributing factors in the occurrence of any mental disorders in pulmonary tuberculosis patients. *European Psychiatry.* 2011;26.

277. Grau LE, Antony S, Brienza R. Veteran patients' perspectives on recent rehospitalization: Qualitative findings. *Journal of General Internal Medicine.* 2017;32(2):S378.

278. Greaves NS, Pooransingh S, Samuels TA, Murphy MM. HIV and Type 2 diabetes: A qualitative exploration of the burden of care experienced and perceived by persons living with multiple co-morbidities in Barbados and Trinidad and Tobago. *West Indian Medical Journal.* 2018;67:38.

279. Harris M, Rhodes T. The changing HCV treatment landscape: New treatments and innovations in modes of service delivery. *Suchtmedizin in Forschung und Praxis.* 2013;15(4):225.

280. Hernández D, Fernández Y, Cardona FT, et al. Understanding drug use and the HIV cascade of care in San Juan, Puerto Rico. *Drug and Alcohol Dependence.* 2015;146:e141.

281. Jacobs E, Zaal F, Alba S. Catastrophic health expenditure and care-seeking practices in Afghanistan: A mixed-methods study. *Tropical Medicine and International Health.* 2017;22:83.

282. Lawson CC, Basseyn S, Friedman AB, Rhodes KV. “There's not really an incentive to do it another way”: Barriers to unscheduled ambulatory care. *Academic Emergency Medicine.* 2015;22(5):S184.

283. Mackinnon S. The health of asylum seekers and refugees in Glasgow: A qualitative study. *Scottish Medical Journal.* 2016;61(4):NP55-NP56.

284. Oelke ND, Schill K, Szostak C, et al. Supporting the mental health needs of adults 50 and over: The importance of integrated community support services. *International Journal of Integrated Care (IJIC).* 2016;16(6):1-2.

285. Sharma K, Cooke C, Howard A, Chater R, Vogler A, Brandt N. Listening to the beneficiaries' voice to optimize the mtm program standardized format. *Consultant Pharmacist.* 2018;33(10):596.

286. Sheridan N, Kenealy T. Consumers with high complex needs co-create a relationship-based approach to care. *International Journal of Integrated Care (IJIC).* 2016;16(6):1-2.

287. Travers J. Access and use of programs of all-inclusive care for the elderly (PACE) for racial/ethnic minority older adults. *Journal of the American Geriatrics Society.* 2019;67:S248.

288. Tsiantou V, Mylona K, Karampli E, Boubouchairopoulou N, Pavi E, Kyriopoulos J. Challenges and Opportunities in The Management of Chronic Diseases During The Economic Crisis In Greece: A Qualitative Approach. *Value in Health.* 2014;17(7):A501-A501.

289. Walsh PG, Duberstein P, Chapman BP, Wittink MN. Discussions of patient stressors and effects on prescribing in primary care: What happens when patients disclose financial concerns. *Journal of General Internal Medicine.* 2018;33(2):86.

290. Yak Leong Chan D, Lian Leng L, Wei Yi T, Lee KH. Identifying communities at high risk of re-admission in Singapore and understanding their needs during transition of care from hospital to home. *International Journal of Integrated Care (IJIC).* 2017;17(3):270-271.

291. Yakovenko I, Clark CM, Hodgins DC, Goghari VM. Schizophrenia and disordered gambling: Qualitative features of dual diagnosis. *Schizophrenia Research.* 2014;153:S188.

292. Bagh J. An awful place for people. *Sygeplejersken / Danish Journal of Nursing.* 2006;106(25-26):56-58.

293. Cummings SAM. A Focused Ethnography of Healthcare Transition among Persistent Asthmatic/Chronic Obstructive Pulmonary Disease (COPD) Adult Pennsylvania Medicaid Beneficiaries. *Focused Ethnography Of Healthcare Transition Among Persistent Asthmatic/Chronic Obstructive Pulmonary Disease (COPD) Adult Pennsylvania Medicaid Beneficiaries.* 2018:1-1.

294. Elkins CL. *Access to healthcare in a free clinic population: What are the barriers?* , ProQuest Information & Learning; 2008.

295. Larson P. The complex self perceptions and relationships of patients who attended free clinics affected their attendance and ability to benefit from care. *Evidence Based Nursing.* 2006;9(2):61-61.

296. Murrey MI. The illness-referral system of low-income older persons living in age-segregated housing. 1980.

297. Nance-Floyd B. *A qualitative study to understand the perception of illness and the decision making process for accessing and utilizing health care for American Indians in Southeastern NC*, ProQuest Information & Learning; 2017.

298. Weiss JAF. *Self-care decision making in clients with diabetes and hypertension*, ProQuest Information & Learning; 1998.

299. Weissman G, Melchior L, Huba G, et al. Women living with substance abuse and HIV disease: medical care access issues. *Journal of the American Medical Women's Association (1972).* 1995;50(3-4):115-120.

300. Zrouki A, Pacciani A, Ledonne G. Care questions and answers. Adherence to therapeutic treatments for chronic illness in the homeless: A qualitative evaluation in the Milanese context. *Ricerca e Pratica.* 2017;33(5):198-214.

301. Acier D, Nadeau L, Landry M. [Process of change in patients with concurrent substance use - mental health problems]. *Sante Mentale au Quebec.* 2007;32(2):59-82.

302. de Abreu KP, Pelegrini AHW, Marques GQ, Lima MADdS. Users' perceptions of urgency and reasons for using the mobile pre-hospital care service. *Revista Gaucha de Enfermagem.* 2012;33(2):146-152.

303. De Mattia Rocha A, Godoy SCB, Carvalho LP, Sa Souza MJB. Management perception about sicken [sic] of the workers from the food service hospital. *Revista Mineira de Enfermagem.* 2007;11(1):53-60.

304. Ka O, Faye A, Mbaye el H, et al. [Living the aging in Senegal Perceptions/representations and coping strategies of persons of age three: results of investigations retrospective]. *Geriatr Psychol Neuropsychiatr Vieil.* 2016;14(1):31-41.

305. Kadio K, Ridde V, Malle SO. [The difficulties of access to health care for indigent living in non-poor households]. *Sante Publique.* 2014;26(1):89-97.

306. Yukawa K, Ishikawa H, Kiuchi T, et al. Economic burden and mental distress in using complementary and alternative medicine, and formal supports by medical professional of Japanese patients with chronic diseases. *Japanese Pharmacology and Therapeutics.* 2017;45(3):345-355.

307. Zerbo R, Drabo KM, Kouanda S, et al. Mise en perspective sociologique de la prise en charge de la co-infection TB/VIH au Burkina Faso = Sociological perspectives on the management of TB/HIV co-infection in Burkina Faso. *Cahiers D'Études et De Recherche Francophone / Santé.* 2010;20(4):195-199.

308. Anstey Watkins JOT, Goudge J, Gomez-Olive FX, Griffiths F. Mobile phone use among patients and health workers to enhance primary healthcare: A qualitative study in rural South Africa. *Social Science & Medicine.* 2018;198:139-147.

309. Arrieta MI, Foreman RD, Crook ED, Icenogle ML. Providing continuity of care for chronic diseases in the aftermath of Katrina: from field experience to policy recommendations. *Disaster med.* 2009;3(3):174-182.

310. Hill KE, Gleadle JM, Pulvirenti M, McNaughton DA. The social determinants of health for people with type 1 diabetes that progress to end-stage renal disease. *Health Expectations.* 2015;18(6):2513-2521.

311. Kern LM, Safford MM, Slavin MJ, et al. Patients’ and Providers’ Views on Causes and Consequences of Healthcare Fragmentation in the Ambulatory Setting: a Qualitative Study. *Journal of General Internal Medicine.* 2019.

312. Remião Luzardo A, Ferreira de Paula Júnior N, Azevedo dos Santos SM, Medeiros M, Bejo Wolkers PC. Repercussions of hospitalization due to fall of the elderly: health care and prevention. *Revista Brasileira de Enfermagem.* 2018;71:763-769.

313. Walters S. *Beyond incredible: The paralympic road to Rio - an ethnographic study*, ProQuest Information & Learning; 2019.

314. Noël PH, Chris Frueh B, Larme AC, Pugh JAJHE. Collaborative care needs and preferences of primary care patients with multimorbidity. 2005;8(1):54-63.
