# Supplementary material for: The experience of financial burden for people with multimorbidity: A systematic review of qualitative research
Source: Health Expect. 2020 Dec 2;24(2):282–95. doi: 10.1111/hex.13166 (PMC8077119; doi:10.1111/hex.13166)
Supplement: Supplementary file 3 — Appendix C [file HEX-24-282-s001.docx]

| Angwenyi^1^ (2018) | Aziz^2^ (2018) | Bair^3^ (2009) | Bardach^4^ (2011) | Bayliss^5^ (2003) |  |
| --- | --- | --- | --- | --- | --- |
| Yes | Yes | Yes | Yes | Yes | Was there a clear statement of the aims of the research? |
| Yes | Yes | Yes | Yes | Yes | Is a qualitative methodology appropriate? |
| Can't tell | Can't tell | Yes | Yes | Yes | Was the research design appropriate to address the aims of the research? |
| Yes | Yes | Yes | Yes | Can't Tell | Was the recruitment strategy appropriate to the aims of the research? |
| Yes | Can't Tell | Yes | Yes | Yes | Was the data collected in a way that addressed the research issue? |
| Can't tell | No | Can't Tell | No | Can't Tell | Has the relationship between researcher and participants been adequately considered? |
| Yes | Yes | Yes | Yes | Yes | Have ethical issues been taken into consideration? |
| Yes | Yes | Can't tell | Yes | Yes | Was the data analysis sufficiently rigorous? |
| Yes | Yes | Yes | Yes | Yes | Is there a clear statement of findings? |

| Beverly^6^ (2011) | Campbell^7^ (2018) | Coventry^8^ (2014) | Dean^9^ (2018) | DiNapoli^10^ (2016) |  |
| --- | --- | --- | --- | --- | --- |
| Yes | Yes | Yes | Yes | Yes | Was there a clear statement of the aims of the research? |
| Yes | Yes | Yes | Yes | Yes | Is a qualitative methodology appropriate? |
| Yes | Yes | Can't tell | Yes | Yes | Was the research design appropriate to address the aims of the research? |
| Yes | Yes | Yes | Yes | Yes | Was the recruitment strategy appropriate to the aims of the research? |
| Yes | Yes | Can't Tell | Yes | Yes | Was the data collected in a way that addressed the research issue? |
| No | Yes | No | No | No | Has the relationship between researcher and participants been adequately considered? |
| Yes | Yes | Yes | Yes | Yes | Have ethical issues been taken into consideration? |
| Can't tell | Yes | Yes | Yes | Yes | Was the data analysis sufficiently rigorous? |
| Yes | Yes | Yes | Yes | Yes | Is there a clear statement of findings? |

| El-Mallakh^11^ (2007) | Elliot^12^ (2007) | Eton^13^ (2012) | Ferguson^14^ (2017) | Fox^15^ (2018) |  |
| --- | --- | --- | --- | --- | --- |
| Yes | Yes | Yes | Yes | Yes | Was there a clear statement of the aims of the research? |
| Yes | Yes | Yes | Yes | Yes | Is a qualitative methodology appropriate? |
| Can't tell | Can't tell | Yes | Yes | Yes | Was the research design appropriate to address the aims of the research? |
| Yes | Yes | Can't Tell | Yes | Yes | Was the recruitment strategy appropriate to the aims of the research? |
| Yes | Yes | Yes | Can't tell | Yes | Was the data collected in a way that addressed the research issue? |
| No | No | No | No | Yes | Has the relationship between researcher and participants been adequately considered? |
| Yes | Yes | Yes | Yes | Yes | Have ethical issues been taken into consideration? |
| Yes | Yes | Yes | Yes | Yes | Was the data analysis sufficiently rigorous? |
| Yes | Yes | Yes | Yes | Yes | Is there a clear statement of findings? |

| Ho^16^ (2017**)** | Hunt^17^ (2012) | Jeon^18^ (2012) | Jeon^19^ (2009) |  |
| --- | --- | --- | --- | --- |
| Yes | Yes | Yes | Yes | Was there a clear statement of the aims of the research? |
| Yes | Yes | Yes | Yes | Is a qualitative methodology appropriate? |
| Yes | Can't tell | Yes | Can't tell | Was the research design appropriate to address the aims of the research? |
| Yes | Yes | Yes | Yes | Was the recruitment strategy appropriate to the aims of the research? |
| Can't Tell | Yes | Yes | Yes | Was the data collected in a way that addressed the research issue? |
| Yes | No | No | No | Has the relationship between researcher and participants been adequately considered? |
| Yes | Yes | Yes | Yes | Have ethical issues been taken into consideration? |
| Yes | Can't tell | Yes | Yes | Was the data analysis sufficiently rigorous? |
| Yes | Yes | Yes | Yes | Is there a clear statement of findings? |

| Kreps^20^ (2011) | Liu^21^ (2019) | Lo^22^ (2016) | Maina^23^ (2019) | Matima^24^ (2018) |  |
| --- | --- | --- | --- | --- | --- |
| Yes | Yes | Yes | Yes | Yes | Was there a clear statement of the aims of the research? |
| Yes | Yes | Yes | Yes | Yes | Is a qualitative methodology appropriate? |
| Yes | Can't Tell | Yes | Yes | Yes | Was the research design appropriate to address the aims of the research? |
| Yes | Can't Tell | Yes | No | Yes | Was the recruitment strategy appropriate to the aims of the research? |
| Yes | Yes | Yes | Can't tell | Can't Tell | Was the data collected in a way that addressed the research issue? |
| No | No | Can't Tell | Can't tell | Can't Tell | Has the relationship between researcher and participants been adequately considered? |
| No | Yes | Yes | Yes | Yes | Have ethical issues been taken into consideration? |
| Can't Tell | Can't Tell | Yes | Yes | Yes | Was the data analysis sufficiently rigorous? |
| Yes | Yes | Yes | Yes | Yes | Is there a clear statement of findings? |

| Mercer^25^ (2010) | Mishra^26^ (2011) | Morgan^27^ (2019) | Naqvi^28^ (2019) | Okombo^29^ (2017) |  |
| --- | --- | --- | --- | --- | --- |
| Yes | Yes | Yes | Yes | Yes | Was there a clear statement of the aims of the research? |
| Yes | Yes | Yes | Yes | Yes | Is a qualitative methodology appropriate? |
| Yes | Yes | Can't Tell | Can't Tell | Yes | Was the research design appropriate to address the aims of the research? |
| Yes | Yes | Yes | No | Yes | Was the recruitment strategy appropriate to the aims of the research? |
| Yes | Yes | Can't Tell | No | Yes | Was the data collected in a way that addressed the research issue? |
| No | Can't Tell | Can't Tell | No | Can't Tell | Has the relationship between researcher and participants been adequately considered? |
| Yes | Yes | Yes | Yes | Yes | Have ethical issues been taken into consideration? |
| Yes | Yes | Yes | No | Can't Tell | Was the data analysis sufficiently rigorous? |
| Yes | Yes | Yes | Yes | Yes | Is there a clear statement of findings? |

| Ortenblad^30^ (2018) | del Pilar^31^ (2012) | Ploeg^32^ (2019) | Sav^33^ (2013) | Schoenberg^34^ (2009) |  |
| --- | --- | --- | --- | --- | --- |
| Yes | Yes | Yes | Yes | Yes | Was there a clear statement of the aims of the research? |
| Yes | Yes | Yes | Yes | Yes | Is a qualitative methodology appropriate? |
| Yes | Yes | Yes | Yes | Yes | Was the research design appropriate to address the aims of the research? |
| Yes | Can't Tell | Yes | Yes | Yes | Was the recruitment strategy appropriate to the aims of the research? |
| Yes | Can't Tell | Yes | Yes | Yes | Was the data collected in a way that addressed the research issue? |
| No | No | Yes | No | Can't Tell | Has the relationship between researcher and participants been adequately considered? |
| Yes | Can't Tell | Yes | Yes | Yes | Have ethical issues been taken into consideration? |
| Yes | Can't Tell | Yes | Yes | Yes | Was the data analysis sufficiently rigorous? |
| Yes | Yes | Yes | Yes | Yes | Is there a clear statement of findings? |

| Shaw^35^ (2018) | Signal^36^ (2017) | Tarasenko^37^ (2011) | Thoman-Touet^38^ (1992) | Tran^39^ (2019) | Tran^40^ (2015) |  |
| --- | --- | --- | --- | --- | --- | --- |
| Yes | Yes | Yes | Yes | Yes | Yes | Was there a clear statement of the aims of the research? |
| Yes | Yes | Yes | Yes | Yes | Yes | Is a qualitative methodology appropriate? |
| Can't tell | Yes | Can't tell | Yes | Yes | Can't tell | Was the research design appropriate to address the aims of the research? |
| Can't Tell | Can't Tell | Yes | Yes | Can't tell | Yes | Was the recruitment strategy appropriate to the aims of the research? |
| Can't Tell | Can't Tell | Yes | Yes | Yes | Yes | Was the data collected in a way that addressed the research issue? |
| No | No | No | Yes | Can't tell | Can't tell | Has the relationship between researcher and participants been adequately considered? |
| No | Yes | Yes | Yes | Yes | Yes | Have ethical issues been taken into consideration? |
| Can't Tell | Yes | Can't Tell | Yes | Yes | Yes | Was the data analysis sufficiently rigorous? |
| No | Yes | Yes | Yes | Yes | Yes | Is there a clear statement of findings? |

| Villena^41^ (2007) | Voils ^42^ (2014) | Volker^43^ 2013 | Warren-Jeanpiere^44^ (2014) | Whittle^45^ (2017) | Whitty^46^ (2014) |  |
| --- | --- | --- | --- | --- | --- | --- |
| Yes | Yes | Yes | Yes | Yes | Yes | Was there a clear statement of the aims of the research? |
| Yes | Yes | Yes | Yes | Yes | Yes | Is a qualitative methodology appropriate? |
| Yes | Can't tell | Yes | Yes | Can't tell | Yes | Was the research design appropriate to address the aims of the research? |
| Yes | Can't tell | Yes | Yes | Can't tell | Yes | Was the recruitment strategy appropriate to the aims of the research? |
| Yes | Can't tell | Yes | Yes | Yes | Yes | Was the data collected in a way that addressed the research issue? |
| Yes | No | No | No | No | No | Has the relationship between researcher and participants been adequately considered? |
| Yes | Yes | Yes | Yes | Yes | Yes | Have ethical issues been taken into consideration? |
| Yes | Yes | Yes | Yes | Yes | Yes | Was the data analysis sufficiently rigorous? |
| Yes | Yes | Yes | Yes | Yes | Yes | Is there a clear statement of findings? |

**References**

1. Angwenyi V, Aantjes C, Kajumi M, De Man J, Criel B, Bunders-Aelen J. Patients experiences of self-management and strategies for dealing with chronic conditions in rural Malawi. PloS one. 2018;13(7).

2. Aziz H, Hatah E, Makmor-Bakry M, Islahudin F, Hamdi NA, Wan IMP. Qualitative exploration of the modifiable factors for medication adherence among subsidised and self-paying patients in Malaysia. BMC health services research. 2018;18(1):1-8.

3. Bair MJ, Matthias MS, Nyland KA, et al. Barriers and facilitators to chronic pain self-management: a qualitative study of primary care patients with comorbid musculoskeletal pain and depression. Pain Medicine. 2009;10(7):1280-1290.

4. Bardach SH, Schoenberg NE, Tarasenko YN, Fleming ST. Rural residents’ perspectives on multiple morbidity management and disease prevention. Journal of Applied Gerontology. 2011;30(6):671-699.

5. Bayliss EA, Steiner JF, Fernald DH, Crane LA, Main DS. Descriptions of barriers to self-care by persons with comorbid chronic diseases. The Annals of Family Medicine. 2003;1(1):15-21.

6. Beverly EA. Incorporating comorbidity, values and preferences into clinical care guidelines designed for older adults living with type 2 diabetes. 2008.

7. Campbell DJT. Assessing the Impact of Financial Barriers on Care and Outcomes for Patients with Cardiovascular-Related Chronic Diseases. 2017.

8. Coventry PA, Fisher L, Kenning C, Bee P, Bower P. Capacity, responsibility, and motivation: a critical qualitative evaluation of patient and practitioner views about barriers to self-management in people with multimorbidity. BMC health services research. 2014;14(1):536.

9. Dean LT, Moss SL, Ransome Y, et al. “It still affects our economic situation”: long-term economic burden of breast cancer and lymphedema. Supportive Care in Cancer. 2019;27(5):1697-1708.

10. DiNapoli EA, Cinna C, Whiteman KL, Fox L, Appelt CJ, Kasckow J. Mental health treatment preferences and challenges of living with multimorbidity from the veteran perspective. International journal of geriatric psychiatry. 2016;31(10):1097-1104.

11. El-Mallakh P. Doing my best: Poverty and self-care among individuals with schizophrenia and diabetes mellitus. Archives of Psychiatric Nursing. 2007;21(1):49-60.

12. Elliott RA, Ross-Degnan D, Adams AS, Safran DG, Soumerai SB. Strategies for coping in a complex world: adherence behavior among older adults with chronic illness. Journal of general internal medicine. 2007;22(6):805-810.

13. Eton DT, de Oliveira DR, Egginton JS, et al. Building a measurement framework of burden of treatment in complex patients with chronic conditions: a qualitative study. Patient related outcome measures. 2012;3:39.

14. Ferguson C, Inglis SC, Newton PJ, Middleton S, Macdonald PS, Davidson PM. Barriers and enablers to adherence to anticoagulation in heart failure with atrial fibrillation: patient and provider perspectives. Journal of clinical nursing. 2017;26(23-24):4325-4334.

15. Fox PL. Diabetes, Depression and Syndemic Suffering among African American Patients: The Intersectionality of Race, Gender and Class 2016.

16. Ho JW, Kuluski K, Im J. “It's a fight to get anything you need”—Accessing care in the community from the perspectives of people with multimorbidity. Health Expectations. 2017;20(6):1311-1319.

17. Hunt LM, Kreiner M, Brody H. The changing face of chronic illness management in primary care: a qualitative study of underlying influences and unintended outcomes. The Annals of Family Medicine. 2012;10(5):452-460.

18. Jeon Y-H, Black A, Govett J, Yen L, McRae I. Private health insurance and quality of life: perspectives of older Australians with multiple chronic conditions. Australian Journal of Primary Health. 2012;18(3):212-219.

19. Jeon Y-H, Essue B, Jan S, Wells R, Whitworth JA. Economic hardship associated with managing chronic illness: a qualitative inquiry. BMC health services research. 2009;9(1):182.

20. Kreps GL, Villagran MM, Zhao X, et al. Development and validation of motivational messages to improve prescription medication adherence for patients with chronic health problems. Patient education and counseling. 2011;83(3):375-381.

21. Liu X-L, Willis K, Fulbrook P, Wu C-J, Shi Y, Johnson M. Factors influencing self-management priority setting and decision-making among Chinese patients with acute coronary syndrome and type 2 diabetes mellitus. European Journal of Cardiovascular Nursing. 2019;18(8):700-710.

22. Lo C, Ilic D, Teede H, et al. The perspectives of patients on health-care for co-morbid diabetes and chronic kidney disease: a qualitative study. PloS one. 2016;11(1).

23. Maina DW. Perspectives of Patients with Comorbidity on the Use of eHealth Technology for Self-Management at Kenyatta National Hospital. 2019.

24. Matima R, Murphy K, Levitt NS, BeLue R, Oni T. A qualitative study on the experiences and perspectives of public sector patients in Cape Town in managing the workload of demands of HIV and type 2 diabetes multimorbidity. PloS one. 2018;13(3).

25. Mercer SW, Siu JY, Hillier SM, et al. A qualitative study of the views of patients with long-term conditions on family doctors in Hong Kong. BMC family practice. 2010;11(1):46.

26. Mishra SI, Gioia D, Childress S, Barnet B, Webster RL. Adherence to medication regimens among low-income patients with multiple comorbid chronic conditions. Health & social work. 2011;36(4):249-258.

27. Morgan SA, Eyles C, Roderick PJ, Adongo PB, Hill AG. Women living with multi-morbidity in the Greater Accra Region of Ghana: a qualitative study guided by the Cumulative Complexity Model. Journal of biosocial science. 2019;51(4):562-577.

28. Naqvi AA, Hassali MA, Aftab MT, Nadir MN. A qualitative study investigating perceived barriers to medication adherence in chronic illness patients of Karachi, Pakistan. Jpma. 2019;69:216.

29. Okombo FA. Racial Ethnic Health Disparities: A Phenomenological Exploration of African American Adults with Diabetes Complications. 2017.

30. Ørtenblad L, Meillier L, Jønsson AR. Multi-morbidity: a patient perspective on navigating the health care system and everyday life. Chronic illness. 2018;14(4):271-282.

31. del Pilar CPM, Cameron BL, Smith DG. Neoliberal-oriented health care system answer to global competition or a threat to health equality for people with chronic illness. Advances in Nursing Science. 2012;35(2):166-181.

32. Ploeg J, Canesi M, Fraser KD, et al. Experiences of community-dwelling older adults living with multiple chronic conditions: a qualitative study. BMJ open. 2019;9(3):e023345.

33. Sav A, Kendall E, McMillan SS, et al. ‘You say treatment, I say hard work’: treatment burden among people with chronic illness and their carers in Australia. Health & social care in the community. 2013;21(6):665-674.

34. Schoenberg NE, Leach C, Edwards W. “It’s a toss up between my hearing, my heart, and my hip”: prioritizing and accommodating multiple morbidities by vulnerable older adults. Journal of health care for the poor and underserved. 2009;20(1):134.

35. Shaw SJ. The pharmaceutical regulation of chronic disease among the US urban poor: an ethnographic study of accountability. Critical public health. 2018;28(2):165-176.

36. Signal L, Semper K, Stairmand J, et al. A walking stick in one hand and a chainsaw in the other: patients’ perspectives of living with multimorbidity. 2017.

37. Tarasenko Y, Schoenberg N. Colorectal cancer screening among rural Appalachian residents with multiple morbidities. 2011.

38. Thoman-Touet SK. A qualitative study of the effect of chronic illness on marital quality. 1992.

39. Tran V-T, Riveros C, Péan C, Czarnobroda A, Ravaud P. Patients’ perspective on how to improve the care of people with chronic conditions in France: a citizen science study within the ComPaRe e-cohort. BMJ quality & safety. 2019;28(11):875-886.

40. Tran V-T, Barnes C, Montori VM, Falissard B, Ravaud P. Taxonomy of the burden of treatment: a multi-country web-based qualitative study of patients with chronic conditions. BMC medicine. 2015;13(1):115.

41. Villena ALD, Chesla CA. Challenges and struggles: Lived experiences of individuals with co-occurring disorders. Archives of Psychiatric Nursing. 2010;24(2):76-88.

42. Voils CI, Sleath B, Maciejewski ML. Patient perspectives on having multiple versus single prescribers of chronic disease medications: results of a qualitative study in a veteran population. BMC health services research. 2014;14(1):490.

43. Volker DL, Becker H, Kang SJ, Kullberg V. A Double Whammy: Health Promotion Among Cancer Survivors with Pre-Existing Functional Limitations. Paper presented at: Oncology nursing forum2013.

44. Warren-Jeanpiere L, Dillaway H, Hamilton P, Young M, Goparaju L. Taking it one day at a time: African American women aging with HIV and co-morbidities. AIDS patient care and STDs. 2014;28(7):372-380.

45. Whittle HJ, Palar K, Ranadive NA, Turan JM, Kushel M, Weiser SD. “The land of the sick and the land of the healthy”: Disability, bureaucracy, and stigma among people living with poverty and chronic illness in the United States. Social Science & Medicine. 2017;190:181-189.

46. Whitty JA, Sav A, Kelly F, et al. Chronic conditions, financial burden and pharmaceutical pricing: insights from Australian consumers. Australian Health Review. 2014;38(5):589-595.
