# Supplementary material for: The experience of financial burden for people with multimorbidity: A systematic review of qualitative research
Source: Health Expect. 2020 Dec 2;24(2):282–95. doi: 10.1111/hex.13166 (PMC8077119; doi:10.1111/hex.13166)
Supplement: Supplementary file 4 — Appendix D [file HEX-24-282-s003.docx]

**GRADE CERQual Qualitative Evidence Profile**

| **Summary of review finding** | **Studies contributing to the review finding** | **Methodological limitations** | **Coherence** | **Adequacy** | **Relevance** | **CERQual assessment of confidence in the evidence** | **Explanation of CERQual assessment** |
| --- | --- | --- | --- | --- | --- | --- | --- |
| **High Costs**  The scale and detail of costs associated with multimorbidity. | | | | | | | |
| **High Costs**  **Direct Costs -** Costs directly related to care | 32 studies contributed to this review finding. | Moderate methodological limitations overall. 27 studies had limitations or lacked details concerning reflexivity, 8 studies had limitations or lacked details concerning recruitment strategies, 10 studies had limitations or lacked details concerning data collection and 7 studies had limitations or lacked details concerning the approach to data analysis. | No or very minor concerns regarding coherence. However, this is a descriptive finding and a broad theme with agreement between relevant studies. Although there was variation in the types of direct costs experienced, the finding reflects the complexity and variation of the data. | No or very minor concerns regarding adequacy. Data is primarily thin however it was a descriptive finding and 32 studies contributed to this finding (financial burden was the main focus of 7 contributing studies). | Minor concerns regarding relevance. Mix of settings, Illnesses, countries (12) and continents (6). A disproportionate number of studies were from the US. | High confidence | 32 studies contributed to the overall review finding. There were moderate methodological limitations. Data from 12 countries across settings. Minor concerns regarding coherence. No or very minor concerns regarding relevance and adequacy. |
| **High Costs**  **Indirect Costs -** Costs indirectly related to care | 14 studies contributed to this review finding. | Moderate methodological limitations overall. 10 studies had limitations or lacked details concerning reflexivity, 2 studies had limitations or lacked details concerning recruitment strategies, 2 studies had limitations or lacked details concerning data collection and 0 studies had limitations or lacked details concerning the approach to data analysis. | Minor concerns regarding coherence. However, this is a descriptive finding with agreement between relevant studies. Some of the phenomena are unique to a small number of studies. | Minor concerns regarding adequacy. Data is primarily thin (financial burden was the main focus of 4 contributing studies), however it was a descriptive finding and 14 studies contributed to this finding. | Moderate concerns regarding relevance. Mix of settings, Illnesses, countries (6) and continents (4). The majority of studies were from the US. | Moderate confidence | 14 studies contributed to the overall review finding. There were moderate methodological limitations. Data from 6 countries across settings. Minor concerns regarding coherence and adequacy. No or very minor concerns regarding relevance. |
| **Financial Coverage**  Financial coverage, from both health insurance (private and public) and government supports, was one of the main moderators of the costs of multimorbidity, determining whether participants experienced costs and how high these costs were | | | | | | | |
| **Financial Coverage**  **Insufficient coverage, insufficient care -** Participants could not afford care because government supports or health insurance only covered healthcare partially, or not at all | 27 studies contributed to this review finding. | Moderate methodological limitations overall. 23 studies had limitations or lacked details concerning reflexivity, 7 studies had limitations or lacked details concerning recruitment strategies, 7 studies had limitations or lacked details concerning data collection and 6 studies had limitations or lacked details concerning the approach to data analysis. | Minor concerns regarding coherence. However, this is a descriptive finding with agreement between relevant studies. The distinction between private and public insurance was not clear in much of the data so broader conclusions were made about insurance regardless of being public or private to ensure adequacy. Sometimes this theme simply refers to high payments despite coverage, and therefore insufficient care is not always applicable. | No or very minor concerns regarding adequacy. Data is primarily thin, however it was a descriptive finding and 27 studies contributed to this finding (financial burden was the main focus of 7 contributing studies). | Minor concerns regarding relevance. Mix of settings, Illnesses, countries (11) and continents (6). A disproportionate number of studies were from the US. | Moderate confidence | 27 studies contributed to the overall review finding. There were moderate methodological limitations. Data from Y countries across settings. Moderate concerns regarding coherence. No or very minor concerns regarding relevance and adequacy. |
| **Financial Coverage**  **Safety-Net -** The financial safety-net that government support and health insurance provided and how this safety-net alleviated financial burden | 12 studies contributed to this review finding. | Moderate methodological limitations overall. 11 studies had limitations or lacked details concerning reflexivity, 2 studies had limitations or lacked details concerning recruitment strategies, 5 studies had limitations or lacked details concerning data collection and 1 studies had limitations or lacked details concerning the approach to data analysis. | Minor concerns regarding coherence. However, this is a descriptive finding with agreement between relevant studies. The distinction between private and public insurance was not clear in much of the data so broader conclusions were made about insurance regardless of being public or private to ensure adequacy. | Moderate concerns regarding adequacy. Data is primarily thin (financial burden was the main focus of 3 contributing studies), however it was a descriptive finding. However, only 12 studies contributed to this finding. | Moderate concerns regarding relevance. Mix of settings, illnesses, countries (6) and continents (4). A disproportionate number of studies were from the US. | Low confidence | 12 studies contributed to the overall review finding. There were moderate methodological limitations. Data from 6 countries across settings. Minor concerns regarding coherence and adequacy. No or very minor concerns regarding relevance. |
| **Financial Coverage**  **Complexity -** Complexity and barriers participants faced when accessing healthcare or reimbursement | 17 studies contributed to this review finding. | Moderate methodological limitations overall. 12 studies had limitations or lacked details concerning reflexivity, 3 studies had limitations or lacked details concerning recruitment strategies, 3 studies had limitations or lacked details concerning data collection and 3 studies had limitations or lacked details concerning the approach to data analysis. | No or very minor concerns regarding coherence. However, this is a descriptive finding with agreement between relevant studies. The distinction between private and public insurance was not clear in much of the data so broader conclusions were made about insurance regardless of being public or private to ensure adequacy. | Moderate concerns regarding adequacy. Data is primarily thin (financial burden was the main focus of 3 contributing studies), however it was a descriptive finding. However, only 17 studies contributed to this finding. | Moderate concerns regarding relevance. Mix of settings and illnesses. Only 4 countries and three continents. The majority of studies were from the US. | Low confidence | 17 studies contributed to the overall review finding. There were moderate methodological limitations. Data from 4 countries across settings. Minor concerns regarding coherence. No or very minor concerns regarding adequacy. Moderate concerns regarding relevance. |
| **Strategies to Manage Costs**  Strategies participants developed to manage high costs | | | | | | | |
| **Strategies to Manage Costs**  **Accessing Informal Supports -** Informal support to manage high costs, including help from family, friends and community resources | 14 studies contributed to this review finding. | Moderate methodological limitations overall. 11 studies had limitations or lacked details concerning reflexivity, 2 studies had limitations or lacked details concerning recruitment strategies, 4 studies had limitations or lacked details concerning data collection and 2 studies had limitations or lacked details concerning the approach to data analysis. | Minor concerns regarding coherence. However, this is a descriptive finding with agreement between relevant studies. Though generally the case, the data were actually quite varied in terms of having informal supports to access. | Minor concerns regarding adequacy. Data is primarily thin (financial burden was the main focus of 3 contributing studies), however it was a descriptive finding and 14 studies contributed to this finding. | Moderate concerns regarding relevance. Mix of settings, illnesses, countries (7) and continents (4). Half of studies were from the US. | Moderate confidence | 14 studies contributed to the overall review finding. There were moderate methodological limitations. Data from 7 countries across settings. Minor concerns regarding adequacy. No or very minor concerns regarding relevance. |
| **Strategies to Manage Costs**  **Making Sacrifices -** Due to high costs, people sometimes chose, or were forced, to make sacrifices such as not accessing healthcare, not adhering to medication regimens, or sacrificing necessities | 31 studies contributed to this review finding. | Moderate methodological limitations overall. 25 studies had limitations or lacked details concerning reflexivity, 5 studies had limitations or lacked details concerning recruitment strategies, 8 studies had limitations or lacked details concerning data collection and 6 studies had limitations or lacked details concerning the approach to data analysis. | Minor concerns regarding coherence. This was primarily a descriptive finding and the finding reflects the variation and complexity of the topic However, this is a descriptive finding with agreement between relevant studies. It was not completely clear that non-adherence or not attending healthcare appointments etc. were ‘sacrifices’ as it was not made explicit by participants that these decisions were to have money for other things such as necessities. | No or very minor concerns regarding adequacy. Data is primarily thin (financial burden was the main focus of 4 contributing studies), however it was a descriptive finding and 31 studies contributed to this finding. | Minor concerns regarding relevance. Mix of settings, illnesses, countries (11) and continents (5). A disproportionate number of studies were from the US. | Moderate confidence | 31 studies contributed to the overall review finding. There were moderate methodological limitations. Data from 11 countries across settings. Moderate concerns regarding coherence. No or very minor concerns regarding relevance and adequacy. |
| **Reduced Wellbeing**  The costs of multimorbidity and the strategies used to manage these costs had a negative impact on people’s wellbeing. This impact on wellbeing manifested itself in many forms such as upset, worry, frustration and stress. | | | | | | | |
| **Reduced Wellbeing** | 16 studies contributed to this review finding. | Moderate methodological limitations overall. 11 studies had limitations or lacked details concerning reflexivity, 2 studies had limitations or lacked details concerning recruitment strategies, 4 studies had limitations or lacked details concerning data collection and 0 studies had limitations or lacked details concerning the approach to data analysis. | Minor concerns regarding coherence. However, this is a descriptive finding with agreement between relevant studies. The word wellbeing was used as it is sufficiently broad to capture a wide range of emotions and experiences. | No or very minor concerns regarding adequacy. Data is primarily thin, however it was a descriptive finding and 16 studies contributed to this finding (financial burden was the main focus of 5 contributing studies). | Moderate concerns regarding relevance. Mix of settings, illnesses, countries (6) and continents (4). Half of studies were from the US. | Moderate  confidence | 16 studies contributed to the overall review finding. There were moderate methodological limitations. Data from 6 countries across settings. Minor concerns regarding coherence. No or very minor concerns regarding relevance and adequacy. |
| **Experiences of People with Multimorbidity of Financial Burden**  The more diseases one has the more costs they have. All other areas of financial burden discussed above are directly related to the costs experienced by the person and therefore it is likely that multimorbidity is exacerbating these areas of financial burden. | 33 studies contributed to this review finding. | Moderate methodological limitations overall. 28 studies had limitations or lacked details concerning reflexivity, 8 studies had limitations or lacked details concerning recruitment strategies, 10 studies had limitations or lacked details concerning data collection and 8 studies had limitations or lacked details concerning the approach to data analysis. | Minor concerns regarding coherence. 13 of the 33 studies directly inferred the relationship between more conditions and more costs. It was clear that high costs were the primary cause of the other themes. | Minor concerns regarding adequacy. Data is primarily thin and this is an explanatory finding, however 33 studies contributed to this finding (financial burden was the main focus of 7 contributing studies). | Minor concerns regarding relevance. Mix of settings, illnesses, countries (12) and continents (6). A disproportionate number of studies were from the US. | Moderate confidence | 33 studies contributed to the overall review finding. There were moderate methodological limitations. Data from 12 countries across settings. Minor concerns regarding coherence and adequacy. No or very minor concerns regarding relevance. |
| **The Effects of Financial Burden on Treatment Burden for People with Multimorbidity and the Effects of Financial Burden on Interactions between People with Multimorbidity and the Healthcare System**  Necessity of work, high costs, adverse clinical outcomes and the complexity of accessing financial coverage effect interactions with the healthcare system (treatment burden). | 42 studies contributed to this review finding. | Moderate methodological limitations overall. 33 studies had limitations or lacked details concerning reflexivity, 9 studies had limitations or lacked details concerning recruitment strategies, 10 studies had limitations or lacked details concerning data collection and 7 studies had limitations or lacked details concerning the approach to data analysis. | Moderate concerns regarding coherence. 4 studies showed a direct relationship between complexity of accessing financial coverage and treatment burden, the relationship was inferred for 13 studies. For all 7 studies that discussed adverse clinical outcomes the negative cycle of adverse clinical outcomes and treatment burden was inferred. | Minor concerns regarding adequacy. Data is primarily thin and this is an explanatory finding, however 42 studies contributed to this finding (financial burden was the main focus of 7 contributing studies). | Minor concerns regarding relevance. Mix of settings, illnesses, countries (12) and continents (6). A disproportionate number of studies were from the US. | Moderate confidence | 42 studies contributed to the overall review finding. There were moderate methodological limitations. Data from 12 countries across settings. Moderate concerns regarding coherence. No or very minor concerns regarding relevance and adequacy. |
