# Supplementary material for: The experience of financial burden for people with multimorbidity: A systematic review of qualitative research
Source: Health Expect. 2020 Dec 2;24(2):282–95. doi: 10.1111/hex.13166 (PMC8077119; doi:10.1111/hex.13166)
Supplement: Supplementary file 5 — Appendix E [file HEX-24-282-s005.docx]

| **Authors, (Year)**  **Country**  **Study setting** | **Qualitative methodology (e.g. Ethnography, narrative, phenomenological, grounded theory)**  **Design (focus group interviews, face-to-face interviews, other)**  **Sampling strategy** | **Data-analysis technique** | **Number of participants and characteristics (age, gender, socioeconomic status, conditions)** | **Operationalization/definition of financial burden** |
| --- | --- | --- | --- | --- |
| Angwenyi^1^ (2018)  Malawi  Members of community home-based care programmes | Study design: Observation, in-depth interviews and four focus-groups  Number of participants in each focus group: NA  Qualitative methodology: Mixed methods  Sampling Strategy: Purposive | A framework approach | **Survey** (observations): 129 participants  Sex N (%): Male 34 (26.4) Female 95 (73.6)  Median age (range):  42 (20–84)  Mean number of conditions (SD): 1.2 (0.4)  Participants with multimorbidity^[[1]](#footnote-1)^ N (%): 19 (14.7)  **Interviews**: 14 participants  Sex N (%): Male 8 (57.1) Female 6 (42.9)  Median age (range):  NA (33–70)  Mean number of conditions (SD): 1.4 (0.5)  Participants with multimorbidity N (%): 6 (42.9)  **Focus-groups**: 31 participants  Sex N (%): Males 11 (35.5) Females 20 (64.5)  Median age (range):  54 (29–73)  Mean number of conditions (SD): 1.3 (0.5)  Participants with multimorbidity N (%): 9 (29.0) | NA |
| Aziz^2^ (2018)  Malaysia  Public clinic, private clinic, public hospital | Study design: Semi-structured face-to-face interviews  Qualitative methodology: NA  Sampling Strategy: Convenience/purposive sampling | Framework analysis approach | 25 participants  Sex N (%): Male 11 (44) Female 14 (56)  Mean age (SD): 52.8 (11.3)  Mean number of conditions (SD): 3.0 (0.7)  Participants with multimorbidity N (%): 25 (100) | NA |
| Bair^3^ (2009)  US  Veteran Affairs (VA) and University primary care clinics | Study design: Four focus group  Number of participants in each group: NA  Qualitative methodology: NA  Sampling Strategy: Random sample of participants in a trial | Thematic content analysis | 18 participants  Sex N (%): Male 7 (39) Female 11 (61)  Mean age (range): 54.8 (27-84)  Mean number of conditions (SD): NA  Participants with multimorbidity N (%): 18 (100) | NA |
| Bardach^4^ (2011)  US  Family and community medicine practices | Study design: In-depth interviews (two for each individual)  Qualitative methodology: Ethnographic approaches  Sampling Strategy: NA | Content analysis | 41 participants  Sex N (%): Male 12 (29.3) Female 29 (70.7)  Mean age (range): 63 (51-77)  Mean number of conditions (Range): 4.7 (2-10)  Participants with multimorbidity N (%): 41 (100) | NA |
| Bayliss^5^ (2003)  US  Primary care | Study design: Semi-structured personal interviews, free listing  Qualitative methodology: NA  Sampling Strategy: NA | Qualitative comparative analysis | 16 participants  Sex N (%): Male 3 (18.7) Female 13 (81.3)  Mean age (SD): NA  Age group (N, %): 31-40 (1, 6.3) 41-50 (3, 18.8) 51-60 (3, 18.8) 61-70 (4, 25) >70 (5, 31.3)  Mean number of conditions (range): 4.3 (3-7)  Participants with multimorbidity N (%): 16 (100) | Financial constraints |
| Beverly^6^ (2011)  US  Community dwelling | Study design: Eight focus groups  Number of participants in each focus group: 3-8  Qualitative methodology: NA  Sampling Strategy: Intensity (purposive) | Thematic and content analysis | 35 participants  Sex N (%): Male 15 (42.9) Female 20 (57.1)  Mean age (range) 74.9 (60-88)  Mean number of conditions (range) 5.6 (3-9)  Participants with multimorbidity N (%): 35 (100) | NA |
| Campbell^7^ (2018)  Canada  General population | Study design: In-depth interviews  Qualitative methodology: Grounded theory  Sampling Strategy: Theoretical or purposeful  sampling strategy | Grounded theory coding  Thematic analysis | 34 participants  Sex N (%): Male 15 (44.1)  Female 19 (55.9)  Mean age (range) 57.9 (31-76)  Mean number of conditions (SD): NA  Participants with multimorbidity N (%): 27 (79.4) | Financial Barriers: Barriers to obtaining  adequate health care, such as necessary prescription medications, due to the costs associated with  these services |
| Coventry^8^ (2014)  England  General practice | Study design: Semi-structured interviews  Qualitative methodology: NA  Sampling Strategy: Purposive | Framework approach | 20 Participants  Sex: NA  Mean age (range): 65.9 (52-88)  Mean number of conditions (range): 2.6 (2-4)  Participants with multimorbidity N (%): 20 (100) | NA |
| Dean^9^ (2018)  US  Trial participants | Study design: Semi-structured interviews  Qualitative methodology: NA  Sampling Strategy: Purposive | Thematic analysis | 40 participants  Sex N (%): Male 0 (0) Female 40 (100)  Mean age (SD): 63 (8)  Mean number of conditions (SD): NA  Participants with multimorbidity N (%): NA | NA |
| DiNapoli^10^ (2016)  US  Primary care | Study design: Semi-structured interview  Qualitative methodology: NA  Sampling Strategy: NA | Thematic analysis | 28 participants  Sex N (%): Male 22 (78.6) Female 6 (21.4)  Mean age (SD): 63.5 (6.3)  Mean number of conditions (SD): NA  Participants with multimorbidity N (%): 28 (100) | NA |
| Elliot^11^ (2007)  US  Community dwelling | Study design: Semi-structured interviews  Qualitative methodology: NA  Sampling Strategy: Purposive | Constant comparison | 20 participants  Sex N (%): Male 8 (40) Female 12 (60)  Mean age (range): 76 (67–90)  Mean number of conditions (range):  5.4 (3–9)  Participants with multimorbidity N (%): 20 (100) | NA |
| El-Mallakh^12^ (2007)  US  Community mental health centre | Study design: Interviews  Qualitative methodology: Grounded theory  Sampling Strategy: Maximum variation sampling | Constant comparison method | 11 participants  Sex N (%): Male 6 (55%)  Female 5 (45%)  Mean age (SD): 50.3 (9.5)  Mean number of conditions (SD): NA Participants with multimorbidity N (%): 11 (100) | Poverty: the deprivation of income, assets, and resources needed to participate in society |
| Eton^13^ (2012)  US  Clinic outpatients | Study design: Semi-structured interviews  Qualitative methodology: NA  Sampling Strategy: NA | Framework analysis | 32 participants  Sex N (%): Male 12 (37) Female 20 (63)  Median age (range): 59.5 (26-85)  Median number of conditions (range): 5 (1-16)  Participants with multimorbidity N (%): NA | NA |
| Ferguson^14^ (2017)  Australia  Tertiary hospital | Study design: Interviews and document analysis  Qualitative methodology: Narrative inquiry  Sampling Strategy: Consecutive patients | Thematic analysis | 144 participants  Sex N (%): Male 94 (65), female 50 (35)  Mean Age (SD): 72 (16.4)  Mean number of conditions (SD): NA  Charlson Comorbidity Index Score (mean, SD) 3.9 ± 2.1  Participants with multimorbidity N (%): 144 (100) | Financial burden |
| Fox^15^ (2018)  US  Outpatient clinic | Study design: Direct observations, clinical interviews, document review  Qualitative methodology: Narrative case study  Framework: Syndemic model  Sampling Strategy: Purposive | Thematic analysis | 6 participants  Sex N (%): Male 3 (50) Female 3 (50)  Mean age (range): 46 (35-56)  Mean number of condition (SD): NA  Participants with multimorbidity N (%): 6 (100) | NA |
| Ho^16^ (2017)  Canada  Continuing care facility | Study design: Secondary analysis on semi- structured interviews  Qualitative methodology: NA  Sampling Strategy: Secondary analysis of previous study which used convenience sample | Exploratory interpretive analysis | 116 Participants  Sex N (%): Male 49 (42) Female 67 (58)  Mean age (SD): NA  Age group (N, %): ≤44 = 13, 45-64 = 52, ≥65 = 46  Mean number of conditions (SD): (n = 109) 5.0 (2.1)  Participants with multimorbidity N (%): 116 (100) | NA |
| Hunt^17^ (2012)  US  Primary care | Study design: Interviews & Observations  Qualitative methodology: NA  Sampling Strategy: Purposive, snowball | Thematic analysis | 70 participants  Sex N (%): Male 33 (47) Female 37 (53)  Mean age (SD): NA  Age-group (N, %): 24-34 (2, 3) 35-44 (9, 13) 45-54 (17, 24) 55-65 (20, 29) >65 (22, 31)  Age range: 32 - 85  Mean number of conditions (SD): NA  Participants with multimorbidity N (%): 41 (58.6) | NA |
| Jeon^18^ (2009)  Australia  Primary care, secondary care, aboriginal health services | Study design: Semi-structured, in-depth  interviews  Qualitative methodology: NA  Sampling Strategy: Purposive sampling | Qualitative  content analysis | 52 participants  Sex N (%): Male 28 (54)  Female 24 (46)  Mean age (SD): NA  Age group N (%): ≤64 years 17 (33)  ≥65 years 35 (67%)  Mean number of conditions (SD): NA  Participants with multimorbidity N (%): 11 (21.2) | Economic hardship in  this paper is defined as perceived economic difficulties that arise as a result of chronic illness and influence the way in which people affected by illness live and manage their conditions. |
| Jeon^19^ (2012)  Australia  Population survey | Study design: Semi-structured interviews  Qualitative methodology: NA  Sampling Strategy: NA | Qualitative content analysis | 40 participants  Sex N (%): Male 17 (42.5) Female 23 (57.5)  Mean age (range) = 70 (53-90)  Mean number of conditions (SD): 6.0 (1.4)  Participants with multimorbidity N (%): 40 (100) | NA |
| Kreps^20^ (2011)  US  Local health clinic | Study design: In-depth  interviews, four focus groups  Number of participants in each focus group: NA  Qualitative methodology: NA  Sampling Strategy: Convenience | Grounded-theory approach (axial coding) | **Interviews***  30 participants  Sex N (%): Male 17 (56.7) Female 13 (43.3)  Mean age (SD): 46 (NA)  Mean number of conditions (SD): NA  Participants with multimorbidity N (%): NA  **Focus groups***  28 participants  Sex N (%): Males 17 (60.7) Female 11 (39.3)  Mean age (SD): 56 (NA)  Mean number of conditions (SD): NA  Participants with multimorbidity N (%): NA  *Unclear if interview and focus group participants were the same participants. | NA |
| Liu^21^ (2019)  China  Inpatient & Outpatient Hospital clinic | Study design: Semi-structured interviews (two for each participants)  Qualitative methodology: NA  Sampling Strategy: Purposive | Content analysis | 21 participants  Sex N (%): Male 18 (85.3) Female 3 (14.7)  Mean age (SD): NA  Mean number of conditions (SD): NA  Participants with multimorbidity N (%): 21 (100) | NA |
| Lo^22^ (2016)  Australia  Tertiary Health-Services | Study design: Twelve focus groups  Number of participants in each group: 3-7  Qualitative methodology: NA  Theoretical framework: Pragmatic worldview  Sampling Strategy: Purposive | Thematic approach | 58 participants  Sex N (%): Male 41 (70.7)  Female 17 (29.3)  Median age (range): 67 (48–84)  Mean number of conditions (SD): NA  Participants with multimorbidity N (%): 58 (100) | NA |
| Maina^23^ (2019)  Kenya  Hospital outpatients | Study design: Interviews  Qualitative methodology: Phenomenology  Sampling Strategy: Purposive | ‘Iterative processes’ | 10 participants  Sex N (%): Male 4 (40) Female 6 (60)  Mean age (SD): 53.9 (10.5)  Mean number of conditions (SD): 2.2 (0.4)  Participants with multimorbidity N (%): 10 (100) | NA |
| Matima^24^ (2018)  South Africa  Two public sector clinics | Study design: In-depth interviews  Qualitative methodology: Phenomenology  Sampling Strategy: Purposive /convenience sampling | Thematic content analysis | 10 participants  Sex N (%): Male 5 (50) Female 5 (50)  Mean age (SD): 46.9 (8.8)  Mean number of conditions (SD): NA  Participants with multimorbidity N (%): 10 (100) | NA |
| Mercer^25^ (2010)  Hong Kong  General population | Study design: One-to-one interviews  Qualitative methodology: NA  Sampling Strategy: Purposively (maximum variation) | Constant comparative method - | 28 participants  Sex N (%): Male 12 (42.9) Female 16 (57.1)  Mean age (range): 52.1 (23-80)  Mean number of conditions (range): 1.4 (1-4)  Participants with multimorbidity N (%): 9 (32.1) | NA |
| Mishra^26^ (2011)  US  Urban University outpatient clinic | Study design: Five focus groups  Number of participants in each focus group: 9-11  Qualitative methodology: Naturalistic phenomenological method (Bronfenbrenner’s ecological model of behaviour)  Sampling Strategy: NA | template analysis | 50 participants  Sex N (%):Male 20 (40)  Female 30 (60)  Age group (N,%):  40–49 (19, 38)  50–59 (17, 34)  60 or older (14, 38)  Mean number of conditions (SD) =4.1 (2.2)  Participants with multimorbidity N (%): 50 (100) | NA |
| Morgan^27^ (2019)  Ghana  Primary care | Study design: In-depth interviews  Qualitative methodology: NA  Framework: Cumulative Complexity Model  Sampling Strategy: Purposive | Thematic analysis | 20 participants  Sex N (%):Male 0 (0) Female 20 (100)  Mean age (SD): 54.8 (10.3)  Mean number of conditions (SD): 2.7 (NA)  Participants with multimorbidity N (%): 20 (100) | NA |
| Naqvi^28^ (2019)  Pakistan  Tertiary care facility | Study design: Interview  Qualitative methodology: Grounded theory  Sampling Strategy: Purposive sampling | Thematic analysis | 16 participants  Sex N (%): Male 8 (50) Female 8 (50)  Mean age (SD): NA  Mean Number of conditions (SD): NA  Participants with multimorbidity N (%): NA | NA |
| Okombo^29^ (2017)  US  General population | Study design: In-depth interviews  Qualitative methodology: Hermeneutical phenomenology  Sampling Strategy: Purpose sampling and snowball sampling | Axial and thematic coding | 6 participants  Sex N (%):Male 1 (16.7) Female 5 (83.3)  Mean Age (SD)= 57.5 (5.6)  Mean number of conditions (SD): 4 (0.8)  Participants with multimorbidity N (%): 6 (100) | NA |
| Ørtenblad^30^ 2018  Denmark  Hospital outpatients | Study design: Observation and in-depth interviews  Qualitative methodology: Ethnography  Sampling Strategy: From focus group study | Inductive approach | 10 participants  Sex N (%): Male 5 (50) Female 5 (50)  Mean age (SD): 51.4 (7.8)  Mean number of conditions (SD): 4.9 (1.5)  Participants with multimorbidity N (%): 10 (100) | NA |
| del Pilar^31^ (2012)  Colombia  Chronic illness support group | Study design: conversations  Qualitative methodology: critical hermeneutic phenomenological approach  Sampling Strategy: NA | Thematic analysis (van Manen) | 5 participants  Sex N (%): Male 2 (40) Female 3 (60)  Mean age (SD): NA  Mean number of conditions (SD)= 2.2 (1.9)  Participants with multimorbidity N (%): NA | NA |
| Ploeg^32^ (2019)  Canada  Community dwelling | Study design: In-depth, semi-structured in-person interviews  Qualitative methodology: Interpretive description (Thorne)  Sampling Strategy: Purposeful sampling | Inductive thematic analysis | 21 participants  Sex N (%): Male 11 (52.4) Female 10 (47.6)  Mean Age (SD): 76.9 (7.4)  Mean number of conditions (SD): 7.4 (2.7)  Participants with multimorbidity N (%): 21 (100) | NA |
| Sav^33^ (2013)  Australia  General population | Study design: Semi-structured in-depth interviews (face to face and telephone)  Qualitative methodology: Interpretive social paradigm  Sampling Strategy: Purposive and snowball  Sampling | Thematic approach and constant  comparison | 85 participants (with chronic illness)  Sex N (%): Male 30 (33) Female 65 (67) (Includes 12 carers)  Mean age 57.2 (13.0) (Includes 12 carers)  Mean number of conditions (SD): NA  Participants with multimorbidity N (%): 75 (88.2) | Financial burden: Feeling concerned and worried about  the financial aspect of their treatment |
| Schoenberg^34^ (2009)  US  General population | Study design: In-depth interviews  Qualitative methodology: Ethnography  Sampling Strategy: Theoretical | Iterative coding | 41 participants  Sex N (%): Male 6 (15%) Female 35 (85%)  Mean age (range): 70.4 (55–90)  Mean number of conditions (SD): NA  Participants with multimorbidity N (%): 41 (100) | NA |
| Shaw^35^ (2018)  US  Health centre | Study design: Ethnographic interviews, 13 focus groups, survey, diary, home visits  Number of participants in each focus group: NA  Qualitative methodology: Mixed methods  Sampling Strategy: A subsample of survey participants | Open-coding method | 71 participants  (Interviews = 35, focus groups = 47, diaries = 15, home visits = 12)  Sex N (%): NA  Mean age (SD) = NA  Mean number of conditions (SD): NA  Participants with multimorbidity N (%): NA | NA |
| Signal^36^ (2017)  New Zealand  Primary care | Study design: Interviews & six focus groups  Number of participants in each focus group: NA  Qualitative methodology: phenomenological perspective  Sampling Strategy: NA | Thematic analysis | 61 participants  (Interviews = 14, focus groups = 47)  Sex N (%): Male 33 (54%) Female 28 (46%)  Mean age (SD): NA  Age groups (N, %) <50 (5, 8), 51–64 (17, 28), 65–74 (20, 33), ≥75 (19, 31)  Mean Number of conditions (SD): NA  Participants with multimorbidity N (%): 61 (100) | NA |
| Tarasenko^37^ 2011  US  Family and community medicine practices | Study design: Semi structured interview  Qualitative methodology:  Mixed methods  Sampling Strategy: Purposive, non-random sampling | ‘Iterative process’ | 41 participants  Sex N (%): Male 12 (29) Female 29 (71)  Mean age (Range): 63 (51-77).  Mean number of conditions (Range): 3 (2-5)  Participants with multimorbidity N (%): 41 (100) | NA |
| Thoman-Touet^38^ (1992)  US  General population | Study design: Open-ended interviews (participants were married couples and were interviewed in pairs, sometime with a child present. One participant was widowed and interviewed individually. Not all participants had a chronic disease)  Qualitative methodology: NA  Sampling Strategy: NA | Content-analysis & domain analysis | 37 adult participants (19 couples)  Sex of adult participants N (%): Male 18 (48.6) Female 19 (51.4)  Mean age (Range): NA (25-84)  Mean number of conditions (SD): NA  Participants with multimorbidity N (%): NA | NA |
| Tran^39^ (2019)  French-speaking countries  General population | Study design: Online questionnaire (open-ended questions)  Qualitative methodology: NA  Sampling Strategy: Recruited from an existing citizen science e-cohort | Thematic analysis | 1,636 participants  Sex N (%): Male 426 (26) Female 1,210 (74)  Mean age (SD): 49 (14.4)  Mean number of conditions (SD): NA Participants with multimorbidity N (%): 834 (51.0) | NA |
| Tran (2015)^40^  Multi-country  General population | Study design: Online survey (open-ended questions)  Qualitative methodology: NA  Sampling Strategy: Snowball/purposive | Content analysis with a grounded theory approach and  automatic textual analysis | 1,053 participants  Sex N (%): Male 285 (27) Female 768 (73)  Median age (IQR): 47 (35–57)  Median number of conditions (IQR): 2 (1–3)  Participants with multimorbidity N (%): 662 (62.9) | Financial impact of the treatment represented both direct costs of healthcare demands and indirect costs associated with treatment (e.g., losing a job, costs of specific foods). |
| Villena^41^ (2007)  US  Community treatment centres  and supportive housing sites | Study design: Interview (all participants interviewed twice)  Qualitative methodology: Interpretive hermeneutic design  Sampling Strategy: Purposive | Interpretive  analysis | 20 participants  Sex N (%): Male 11 (55) Female 9 (45)  Mean age (SD): 51 (NA)  Mean number of conditions (SD): NA  Participants with multimorbidity N (%): 12 (60) | NA |
| Voils^42^ (2014)  US  Outpatient Veterans Affairs Medical Centre | Study design: Three focus groups  Number of participants in each focus group: 6-9  Qualitative methodology: NA  Sampling Strategy: Sampled from existing quantitative study | Content analysis | 23 participants  Sex N (%): Male 21 (91.3) Female 2 (8.7)  Mean Age (SD): 65.4 (11.1)  Mean number of conditions (SD): 2.3 (0.5)  Participants with multimorbidity N (%): 23 (100) | NA |
| Volker^43^ 2013  US  Cancer study participants | Study design: Four focus group  Number of participants in each focus group: NA  Qualitative methodology: Descriptive  Sampling Strategy: From existing study | Content analysis | 19 Participants  Sex N (%): Male 0 (0) Female 19 (100)  Mean age (SD): 59.5 (9.6) Mean number of conditions (SD): NA  Participants with multimorbidity N (%): 19 (100) | NA |
| Warren-Jeanpiere^44^ (2014)  US  HIV study participants | Study design: Five focus groups  Number of participants in each focus group:  Qualitative methodology: N.A.  Sampling Strategy: Purposive sampling from existing study | Constant-comparison technique | 23 Participants  Sex N (%): Male 0 (0) Female 23 (100)  Mean Age (SD): 56.9 (3.6)  Mean number of conditions (SD): 3.4 (1.4)  Participants with multimorbidity N (%): 23 (100) | NA |
| Whittle^45^ (2017)  US  General population (receiving food assistance) | Study design: Semi-structured in-depth interviews  Qualitative methodology: NA  Theoretical framework: structural stigma  Sampling Strategy: NA | Thematic content analysis | 64 participants  Sex N (%): Male 44 (69) Female 17 (27) Transwoman 1 (2) Other 2 (3)  Age (N, %): 35–49 (10, 16) 50–64 (45, 70) 65+ (9, 14)  Mean number of conditions (SD): 1.2 (0.4)  Participants with multimorbidity N (%): 11 (17.2) | NA |
| Whitty^46^ (2014)  Same data as Sav (2013) | Study design: Same data as Sav (2013)^33^  Qualitative methodology: NA  Sampling Strategy: Same data as Sav (2013 | Constant comparison method | Same data as Sav (2013) | NA |

1. Multimorbidity is the presence of two or more conditions in an individual. [↑](#footnote-ref-1)
